# Beyond GLP-1 Agonists: Plant-Derived Bioactive Compounds as Adjunctive Strategies for Obesity Management

**DOI:** 10.3390/nu18142266

**Published:** 2026-07-10

**Authors:** Aurelian Vasile, Andrei Cristian Anghel, Teodor Ioan Trasca, Alina Ortan

**Affiliations:** 1 Faculty of Agriculture, University of Agronomic Sciences and Veterinary Medicine of Bucharest, 59 Marasti Blvd, District 1, 011464 Bucharest, Romania; aurelian.vasile@agro.usamv.ro; 2 Faculty of Biotechnology, University of Agronomic Sciences and Veterinary Medicine of Bucharest, 59 Marasti Blvd, District 1, 011464 Bucharest, Romania; andrei.anghel@ibna.ro; 3 National Research-Development Institute for Animal Biology and Nutrition (IBNA), 1 Calea Bucuresti, 077015 Balotesti, Romania; 4 Faculty of Animal Production Engineering and Management, University of Agronomic Sciences and Veterinary Medicine of Bucharest, 59 Marasti Blvd, District 1, 011464 Bucharest, Romania; 5 Faculty of Land Reclamation and Environmental Engineering, University of Agronomic Sciences and Veterinary Medicine of Bucharest, 59 Marasti Blvd, District 1, 011464 Bucharest, Romania

**Keywords:** obesity, GLP-1 receptor agonists, polyphenols, plant-derived bioactive compounds, incretin therapy, narrative review, weight management

## Abstract

*Background:* Obesity has reached epidemic proportions, affecting over one billion adults worldwide. While incretin-based pharmacotherapies—GLP-1 receptor agonists (semaglutide) and dual GIP/GLP-1 agonists (tirzepatide)—have transformed obesity treatment, their use remains limited by high costs, adverse effects, restricted eligibility, and rapid weight regain following discontinuation. Plant-derived bioactive compounds represent a promising complementary approach to address these gaps. *Objective:* This narrative review synthesizes clinical trial evidence on plant-based interventions for obesity management and examines their potential role as adjunctive strategies before, during, and after incretin-based pharmacotherapy. *Methods:* A literature search was conducted in PubMed, Scopus, and Web of Science (2012–2026), prioritizing randomized controlled trials in adults with overweight or obesity reporting at least one obesity-related metabolic outcome. Sixty-one studies were selected and classified by efficacy tier based on the magnitude and breadth of observed clinical effects. *Results:* The strongest evidence supports polyphenol-rich dietary patterns, particularly the green-Mediterranean diet, producing significant reductions in body weight, visceral fat, and cardiometabolic risk markers. Specific extracts—including curcumin, bergamot polyphenols, and *Lippia citriodora*/*Hibiscus sabdariffa* combinations—demonstrated clinically meaningful metabolic improvements. Isolated high-dose resveratrol and several single-compound interventions showed limited benefit, largely attributable to poor bioavailability. The most effective compounds acted through multiple pathways, including AMPK activation, gut microbiota modulation, and appetite hormone regulation. *Conclusions:* Plant-derived bioactive compounds offer a safe, accessible adjunctive strategy for obesity management, particularly relevant for patients ineligible for or discontinuing pharmacotherapy. Future trials should directly evaluate plant-polyphenol combinations alongside GLP-1 receptor agonists.

## 1. Introduction

Obesity was initially viewed by the medical community as an individual’s lack of willpower to refrain from eating. After extensive research, this paradigm changed, and obesity began to be regarded as a hormonal dysfunction. This new paradigm became firmly established with the introduction of pharmacological treatments for obesity, such as GLP-1 mimetics therapies, particularly semaglutide and tirzepatide. GLP-1 receptor agonists were originally developed for the treatment of type 2 diabetes and were subsequently approved for obesity management. Their mechanism of action involves stimulating insulin secretion, prolonging the transit time of chyme through the digestive tract, and orchestrating cellular signaling pathways associated with satiety in a manner that reduces appetite. These processes produce remarkable results, as individuals suffering from obesity can achieve outcomes comparable to those obtained through bariatric surgical intervention. However, GLP-1 mimetics-based therapies have certain limitations, including high production costs, supply shortages that cannot meet increasing demand, injectable administration, loss of muscle mass during long-term treatment, gastrointestinal adverse effects, and the return of patients to an obese status after treatment discontinuation. To address these limitations, researchers have increasingly focused on identifying natural therapeutic agents derived from plants, with the aim of overcoming some disadvantages of GLP-1 mimetics therapies. Their main proposed mechanism involves the stimulation of endogenous GLP-1 secretion, potentially supporting a more sustainable therapeutic response. Therefore, this paper presents findings from recent clinical studies in which individuals with obesity consumed different plants or plant extracts and either exhibited or failed to exhibit metabolic improvements, thereby identifying the most promising plant-based therapeutic strategies. At the same time, this study explores the potential synergy between pharmacological agents and plant consumption as a strategy for achieving more effective outcomes in the treatment of obesity.

Human metabolism evolved to store energy and maintain body weight during times of food scarcity [1,2]. At present, the modern environment provides widespread access to hypercaloric, ultra-processed foods, making overconsumption a complex challenge driven by deeply rooted evolutionary, genetic, and environmental factors rather than merely poor willpower [3,4,5].

Current approaches to treating obesity involve the administration of artificial glucagon-like peptide-1 (GLP-1), which plays a role in delaying the passage of food from the stomach to the intestine and in reducing insulin resistance, while also inducing the sensation of satiety [6,7]. However, not everyone is eligible for this therapy; clinical guidelines generally restrict these medications to individuals classified as obese or overweight with existing weight-related comorbidities, frequently excluding those only in the early stages of weight gain [8]. Furthermore, access to these drugs is often hindered by high costs and adverse gastrointestinal side effects [9,10].

At the same time, after the end of the GLP-1 mimetics administration period, sustaining weight loss also requires long-term lifestyle changes, as clinical trials consistently demonstrate that discontinuing these medications results in rapid and substantial weight regain [11,12].

Plant polyphenols may stimulate endogenous GLP-1 secretion, which can support appetite regulation, satiety, and glycemic control [2,4]. A supplement that combines polyphenolic extracts from *Lippia citriodora* and *Hibiscus sabdariffa* significantly increased circulating GLP-1 levels over two months [2,4]. This combination may also help counteract the “energy gap” during calorie restriction, when weight loss usually reduces energy expenditure and increases hunger, and it appears to act by increasing GLP-1, reducing ghrelin, and improving satiety [2].

Stilbene polyphenols, which are common in grapes, can also stimulate GLP-1 secretion by activating the SIRT1 pathway, downregulating PPARγ, and increasing GLP-1 release, which contributes to improved glycemic control [13]. Anthocyanins, found in berries, red fruits, black soybeans, and purple sweet potatoes, affect appetite-related hormones such as GLP-1, GIP, and CCK, and they promote satiety and reduce caloric intake [14]. Furthermore, grape seed proanthocyanidins may raise circulating GLP-1 levels and delay gastric emptying, which can prolong satiety [15].

Polyphenols may also stimulate GLP-1 secretion indirectly, through a prebiotic effect on the gut microbiome [13,16]. Unabsorbed polyphenols that reach the large intestine promote beneficial bacteria, which ferment fibers and polyphenols into short-chain fatty acids (SCFAs) such as acetate, propionate, and butyrate [13,14]. These SCFAs can stimulate intestinal enteroendocrine L-cells, partly through receptors such as FFAR2, leading to the secretion of GLP-1 and PYY [16,17]. Moreover, the resulting GLP-1 and PYY response helps regulate energy balance by increasing insulin secretion, inhibiting glucagon release, and suppressing appetite [16].

In this context, natural bioactive compounds, such as plant-derived polyphenols, represent a promising and safe adjunct strategy for weight management [4,15].

Therefore, this study aims to identify the most effective plant-based strategies for combating obesity based on clinical trials, while also examining how the intake of plants or plant-derived extracts can complement pharmacological treatments to enhance therapeutic efficacy and mitigate the rebound weight gain that frequently follows the cessation of anti-obesity drugs [4,14,15,17,18].

Obesity is a chronic disease characterized by an excessive accumulation of body fat that poses significant health risks [1,3,19]. Although it is clinically defined by a body mass index (BMI) of 30 kg/m^2^ or higher, BMI is increasingly recognized as an imperfect and limited measure [3,20]. Over time, the understanding of obesity has changed substantially, shifting from a simplistic view centered on overeating and laziness to the recognition that it is a complex, multifactorial condition [21].

Plant-derived compounds, known as phytoactives, have been used for centuries in traditional medical systems, particularly in Asia and the Mediterranean. These practices helped shape modern Complementary and Alternative Medicine (CAM) and continue to inform pharmacological research [2,4,15,16,20,22,23,24,25].

In Indian and Sri Lankan traditions, especially Ayurveda and Siddha, medicinal plants have long been used to regulate body weight and support metabolic health. Moreover, Ayurveda employs herbal formulations such as Triphala, Garcinia cambogia, and Cassia fistula for body weight management and central obesity, and modern clinical studies suggest that these preparations may be effective. Furthermore, the Sri Lankan Siddha system relies on complex multi-herb formulations, including Piramehachchanthanaathiyennai, which contains phytoactives derived from 54 plant species across 40 families [2,4,15,16,20,22,23,24,25].

Natural bioactive compounds, including polyphenols, flavonoids, alkaloids, and omega-3 fatty acids, may help manage complex metabolic disorders such as obesity and diabetes through pleiotropic, multi-target actions rather than a single specific effect [13]. These compounds modulate multiple interconnected genetic, cellular, and physiological pathways simultaneously [26], with a central mechanism being the regulation of energy metabolism and mitochondrial function through the AMPK/SIRT1/PGC-1α signaling cascade, which promotes mitochondrial biogenesis, increases fatty acid β-oxidation, and protects mitochondrial integrity from oxidative damage [15,26,27].

Specific compounds such as resveratrol, capsaicin, and green tea catechins further promote the browning of white adipose tissue and enhance non-shivering thermogenesis [15,18]. In addition, these compounds inhibit adipogenesis and lipid accumulation by reducing de novo lipogenesis and enhancing lipolysis, thereby promoting the use of stored fat as an energy source [4,15,17,18].

Bioactive compounds reinforce anti-inflammatory and antioxidant defenses by inhibiting NF-κB and MAPK signaling, lowering pro-inflammatory cytokines such as TNF-α, IL-6, and IL-1β, promoting the shift in adipose tissue macrophages from the pro-inflammatory M1 to the anti-inflammatory M2 phenotype, and countering oxidative stress through ROS neutralization and activation of the Nrf2 pathway to increase endogenous antioxidant enzymes such as SOD and CAT [17,26,28,29]. Furthermore, these compounds improve gut health by modulating the microbiome composition, lowering the Firmicutes/Bacteroidetes ratio, increasing beneficial bacteria, and raising short-chain fatty acid production, which collectively strengthens the intestinal barrier, improves insulin sensitivity, and suppresses appetite [4,13,15,17].

**Figure 1:**
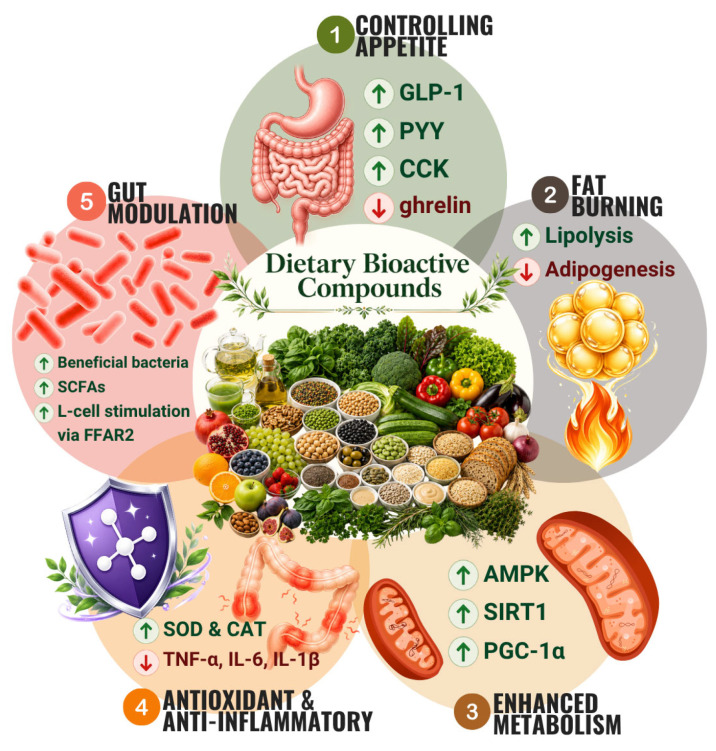
Proposed multi-target mechanisms of action of dietary bioactive compounds in obesity management. Plant-derived bioactive compounds act through five convergent pathways: (1) controlling appetite via modulation of GLP-1, (glucagon-like peptide-1), PYY (peptide YY), and CCK (cholecystokinin) secretion and suppression of ghrelin; (2) promoting fat burning by enhancing lipolysis and inhibiting adipogenesis; (3) enhancing energy metabolism through activation of the AMPK (adenosine monophosphate-activated protein kinase)/SIRT1 (sirtuin 1)/PGC-1α (peroxisome proliferator-activated receptor gamma coactivator 1-alpha) signaling axis; (4) reinforcing antiox-idant and anti-inflammatory defenses by upregulating SOD (superoxide dismutase) and CAT (catalase) and downregulating TNF-α (tumor necrosis factor-alpha), IL-6 (interleukin-6), and IL-1β (interleukin-1 beta); and (5) modulating gut microbiota composition by increasing beneficial bacteria, short-chain fatty acid (SCFA; short-chain fatty acid) production, and L-cell (enteroendocrine L-cell) stimulation via FFAR2 (free fatty acid receptor 2).

Figure 1 summarizes these convergent mechanisms through which dietary bioactive compounds exert anti-obesity effects.

In sarcopenic obesity, bioactive compounds help preserve muscle mass by activating the Akt/mTOR pathway to promote protein synthesis while suppressing FOXO3a, MuRF1, and Atrogin-1 to reduce muscle protein breakdown [26,27]. They also improve glycemic control by inhibiting digestive enzymes such as α-amylase, α-glucosidase, and pancreatic lipase and by enhancing insulin signaling through the PI3K/Akt pathway [4,15,17,18].

## 2. Materials and Methods

### 2.1. Review Design

This review was conducted as a narrative review of clinical evidence on plant-derived bioactive compounds in the context of obesity management. A narrative review design was selected to facilitate the integration of heterogeneous clinical trial data across diverse botanical categories, study populations, and intervention protocols, with the aim of identifying clinically meaningful patterns rather than pooling quantitative effect estimates. The review was structured in accordance with the Scale for the Assessment of Narrative Review Articles (SANRA) criteria.

### 2.2. Literature Search Strategy

A systematic literature search was performed in three electronic databases—PubMed/MEDLINE, Scopus, and Web of Science—covering publications from January 2012 to May 2026. The search was conducted in May 2026 using combinations of the following terms:

*For plant-based interventions:* (“polyphenol” OR “phytochemical” OR “plant extract” OR “botanical” OR “nutraceutical” OR “flavonoid” OR “resveratrol” OR “curcumin” OR “EGCG” OR “green tea” OR “Mediterranean diet” OR “plant-based diet”) AND (“obesity” OR “overweight” OR “body weight” OR “adiposity” OR “visceral fat” OR “BMI”) AND (“clinical trial” OR “randomized controlled trial” OR “RCT”).

*For incretin pharmacotherapy context*: (“GLP-1” OR “glucagon-like peptide-1” OR “semaglutide” OR “tirzepatide” OR “incretin” OR “GLP-1 receptor agonist”) AND (“obesity” OR “weight loss” OR “weight management”).

Additional records were identified through manual screening of reference lists of included studies and relevant review articles. The grey literature was not systematically searched.

### 2.3. Eligibility Criteria

Studies were included based on the following pre-defined criteria (Table 1), organized using the PICO framework:

### 2.4. Study Selection and Data Extraction

Records retrieved from the three databases were deduplicated and screened by title and abstract for relevance. Full-text articles were then assessed against the eligibility criteria. For each included study, the following data were extracted: study design, country, sample size, participant characteristics (age, sex, BMI range, relevant comorbidities), intervention details (compound, dose, formulation, duration), comparator, key obesity-related outcomes, and reported adverse events.

### 2.5. Efficacy Classification and Ranking

Given the heterogeneity of interventions and outcome measures across included studies, a standardized pooled meta-analysis was not feasible. Instead, included studies were classified into five empirically defined efficacy tiers, based on the nature and magnitude of obesity-related effects reported:

- HIGH: Statistically significant direct reduction in body weight and/or body fat mass, accompanied by improvements across multiple cardiometabolic endpoints (e.g., insulin resistance, lipid profile, adipokines, hepatic or visceral fat).
- MODERATE: Statistically significant improvement in body composition (weight, fat mass, or waist circumference) of smaller magnitude, or consistent metabolic improvements without a primary weight loss effect.
- LOW–MODERATE: Limited, indirect, or short-term obesity-related effects, including favorable changes in mechanistic biomarkers (e.g., fat oxidation, energy expenditure, postprandial glucose) without demonstrable change in body composition.
- LOW: Effects confined to surrogate biomarkers (e.g., inflammatory cytokines, oxidative stress markers, acute incretin responses) with no change in weight, fat mass, or primary metabolic endpoints.
- NULL/NEGATIVE: No statistically significant obesity-related effect observed on any pre-specified endpoint, or emergence of unfavorable outcomes.

Tier assignment was performed independently by two reviewers and confirmed by consensus. Within each tier, studies were ordered by the breadth of endpoints improved and by clinical relevance of the primary outcome.

### 2.6. Narrative Synthesis

Included studies were narratively synthesized and organized thematically by botanical category (e.g., polyphenol-rich dietary patterns, resveratrol-based interventions, green tea and EGCG, curcumin, Hibiscus extracts, fruits and berries, cereals and nuts, spices, and marine functional foods). For each category, clinical outcomes were described in relation to reported mechanistic pathways where available. The role of plant-based interventions was then contextualized within three clinical scenarios relevant to incretin-based pharmacotherapy: (i) patients ineligible for drug treatment; (ii) adjunctive use during pharmacological treatment; and (iii) maintenance of weight loss following treatment discontinuation.

## 3. Results

### 3.1. Clinical Evidence for Efficacy of Plants and Plant-Derived Compounds

Dietary interventions rich in plants and plant-derived compounds, particularly polyphenols, have emerged as promising therapeutic strategies for the management of chronic diseases such as obesity, type 2 diabetes, and cardiovascular disease [31,32,33].

Found abundantly in fruits, vegetables, and other botanical sources, polyphenols are secondary metabolites with important antioxidant and anti-inflammatory properties [31,32,34]. Moreover, evidence from randomized controlled trials suggests that these natural bioactive compounds can modulate lipid and glucose metabolism, improve insulin sensitivity, and reduce cardiometabolic risk factors (Table 2) [33,35,36].

### 3.2. Dietary Patterns and Gut Microbiota Modulation

The green-Mediterranean (Green-MED) diet (highly effective). The green-Mediterranean (green-MED) diet heavily restricts meat and increases plant-based foods compared to the standard Mediterranean diet. A specific calorie-restricted protocol adds walnuts, green tea, and the Mankai aquatic plant to significantly boost daily polyphenol and plant protein intake. Furthermore, following this diet for 6 to 18 months causes substantial weight loss and lowers overall cardiometabolic risk [38]. Additionally, the plan nearly doubles the loss of both intrahepatic fat and visceral adipose tissue compared to standard healthy guidelines, greatly reducing the prevalence of non-alcoholic fatty liver disease [37,39]. These significant fat reductions occur because plant-based polyphenols elevate urolithin A, increase fatty acid oxidation, enhance thermogenesis, reduce liver cell damage, and induce the browning of white adipose tissue [37,39]. Also, strictly limiting red meat prevents the activation of oxidative stress and proinflammatory cytokines [92]. Moreover, the green-MED diet drives favorable structural and functional improvements in the gut microbiome. Specifically, increased abundances of Prevotella and Ruminococcaceae bacteria, enhance amino acid degradation pathways and mediate liver fat reduction independently of general weight loss [37,38].

Autologous Fecal Microbiota Transplantation (aFMT) (moderately effective). Another highly effective strategy for combating obesity is autologous fecal microbiota transplantation (aFMT). This approach involves collecting an individual’s own fecal microbiota during a biologically favorable period, such as the peak of diet-induced weight loss, and reintroducing it at a later time point. Compared to donor-derived FMT, aFMT offers a notable safety advantage by eliminating the risk of interindividual pathogen transmission [59,60]. In obesity and metabolic health, aFMT has been investigated as a strategy to limit weight regain and the return of cardiometabolic risk factors after weight loss [59]. Its effectiveness appears to depend on the diet followed during the initial weight-loss phase, as the DIRECT-PLUS trial showed that aFMT attenuated weight regain, waist circumference increase, and insulin rebound only in participants who had followed a high-polyphenol green-Mediterranean diet [59]. This diet included green tea and Mankai and restricted red and processed meat, and it appeared to create a more favorable microbiome for later transplantation [37,59]. Furthermore, the benefit of aFMT was linked to the preservation of diet-induced microbiome changes, including beneficial bacteria such as Alistipes putredinis and Bacteroides vulgatus and microbial pathways related to sugar transport [59,60]. Moreover, response to aFMT was associated more strongly with changes in the non-core, low-abundance microbiota than in the core microbiota, suggesting that rare bacterial populations play an important role in sustained weight maintenance [60]. aFMT was administered as 100 frozen oral capsules, or 100 g processed material, over 6 months and was reported to be safe, with high adherence and no severe adverse events or gastrointestinal symptoms compared with placebo [59]. Additionally, animal studies supported these findings by showing that aFMT collected after a Mankai-supplemented weight-loss diet reduced weight regain and improved glucose tolerance during a later high-fat diet challenge [59].

Prebiotic and Polyphenol Combinations (low efficacy). Another example through which gut microbiota modulation contributes significantly to improving metabolic health is provided by a clinical trial in which patients were orally administered a blend of prebiotics and polyphenols, including inulin, oat β-glucan, blueberry anthocyanins, and blueberry polyphenols, at a specific dose consisting of 4 g of inulin and 2.5 g of oat β-glucan, providing 8.8 g of total fiber, along with a blueberry extract equivalent to two cups of whole blueberries without the sugar [75]. Administration of this gastrointestinal microbiome modulator twice daily for four weeks resulted in improved blood glucose tolerance and increased subjective satiety, likely because the viscosity generated by the fiber components stimulates satiety signals by delaying gastric emptying, while the polyphenols inhibit starch-digestion enzymes [75].

Mediterranean Fruit and Vegetable Polyphenol Extract (moderately effective). Similarly, a polyphenol extract derived from fruits and vegetables commonly consumed in the Mediterranean diet, including grapefruit, grape, guarana seed, green tea, and black carrot, provides a range of bioactive compounds such as flavonoid polyphenols, natural methylxanthines from guarana seed, and vitamin B3. Administration of 900 mg/day of this extract for 16 weeks resulted in improved health-related quality of life, reduced body fat mass, and increased physical activity. These effects are likely attributable to improved vascular function through antioxidative and anti-inflammatory properties, potential inhibition of pancreatic lipase, and enhanced lipolysis [22].

### 3.3. Resveratrol-Based Interventions

Resveratrol Combined with a Lipstatin Derivative (highly effective). Resveratrol is a naturally occurring polyphenol of the stilbene class found mainly in the skin of grapes, berries, peanuts, and red wine and it has attracted considerable scientific and commercial interest owing to its proposed ability to mimic the health benefits of calorie restriction [70,85,91]. When combined with a synthetic derivative of lipstatin, a natural product isolated from Streptomyces toxytricini that inhibits gastric and pancreatic lipases and thereby reduces intestinal fat absorption and overall caloric intake, these two agents produce clinically significant effects on body weight, fasting glucose, and glycosylated hemoglobin (HbA1c) levels [40]. Specifically, administration of 120 mg of the lipstatin derivative combined with 100 mg of resveratrol three times daily for six months, together with an energy-reduced diet, resulted in significant weight loss and decreased body mass index, while also reducing waist circumference, fat mass, triglycerides, leptin, and the leptin/adiponectin ratio, likely through a synergistic mechanism in which the lipstatin derivative lowers fat absorption by inactivating lipases, whereas resveratrol prevents lipid accumulation and enhances fatty acid oxidation through pathways involving AMPK, Sirt1, and PGC-1α [40].

Resveratrol Combined with Myo-Inositol (moderately effective). Myo-inositol, a carbocyclic sugar and vital structural component of cellular membrane phospholipids, serves as a precursor to inositol triphosphate and acts as a second messenger in the regulation of hormones such as insulin and follicle-stimulating hormone (FSH) [51]. Within intracellular signal transduction, the binding of insulin to its receptors triggers a myo-inositol-dependent pathway that facilitates the oxidative metabolism of glucose [51]. Clinical observation has shown that the dual administration of 1000 mg of resveratrol and 1000 mg of myo-inositol twice daily for 12 weeks leads to significant reductions in weight, BMI, and waist–hip ratio among women with polycystic ovary syndrome; this intervention improves glucose homeostasis via SIRT1 activity, while myo-inositol concurrently functions as a second messenger to promote oxidative glucose metabolism [51].

Resveratrol Combined with Curcumin: Acute Postprandial Effects (low efficacy). Administration of a single dose of 200 mg resveratrol and 100 mg curcumin, monitored over a 6 h period, significantly reduced the cumulative postprandial response of soluble vascular cell adhesion molecule-1 (sVCAM-1), although the overall anti-inflammatory effects remained limited, likely because of the relatively low acute dose and poor bioavailability; nevertheless, the selective reduction in sVCAM-1 was likely mediated by circulating plasma metabolites [79].

Resveratrol Alone: Adipose Tissue Effects (low–moderate efficacy). Similarly, administration of 150 mg/day of resveratrol for 30 days significantly decreased abdominal subcutaneous adipocyte size and was accompanied by a 41% reduction in ACE2 gene expression and a 31% reduction in leptin expression in the abdominal subcutaneous adipose tissue of males with obesity, while downregulation of Wnt and Notch signaling pathways together with upregulation of cell cycle genes suggested enhanced adipogenesis, and increased lysosomal and phagosomal pathway activity indicated alternative lipid breakdown [70,71].

Resveratrol: Studies with Limited or Negative Outcomes (null/negative efficacy). In contrast, administration of 75 mg/day of resveratrol for 12 weeks produced no changes in body composition, resting metabolic rate, plasma lipids, inflammatory markers, or insulin sensitivity, and it also failed to affect its proposed molecular targets in skeletal muscle or adipose tissue, suggesting that any metabolic benefit of resveratrol may be more relevant in obese individuals than in the glucose-tolerant women studied [85] (null/negative efficacy). Likewise, administration of 500 mg of resveratrol three times daily (total 1.5 g/day) for 6 months resulted in no changes in basal or insulin-mediated very low-density lipoprotein triglyceride secretion, clearance, or oxidation rates, while palmitate turnover and intrahepatic triglyceride content also remained unaffected, indicating that the reduced insulin sensitivity associated with NAFLD and obesity may blunt the effects of resveratrol [90] (null/negative efficacy). Similarly, another study found no significant improvements in plasma transaminases, liver lipid content, histological features, or insulin sensitivity, with low bioavailability proposed as the likely explanation [89] (negative efficacy). In addition, administration of 3000 mg of resveratrol for eight weeks did not reduce insulin resistance, hepatic steatosis, or abdominal fat distribution and instead increased hepatic stress, as reflected by elevated liver enzymes, suggesting that its extensive conjugation and poor bioavailability may function as a protective mechanism against high-dose exposure in clinical conditions marked by dysregulated metabolic pathways [91].

Resveratrol has shown beneficial effects in several studies, especially in individuals with metabolic dysfunction. In patients with type 2 diabetes, moderate doses such as 200 mg/day reduced fasting plasma glucose, HbA1c, insulin levels, insulin resistance measured by HOMA-IR, and inflammatory markers such as TNF-α and IL-6 [29]. Similarly, in obese men, 150 mg/day improved mitochondrial efficiency, decreased resting metabolic rate, lowered triglycerides, and reduced systolic blood pressure [4,70]. Moreover, resveratrol inhibited adipocyte differentiation, reduced fat accumulation, and promoted a higher proportion of smaller and healthier adipocytes [14,70]. It also showed cardiovascular and neurological potential by attenuating atherosclerosis through gut microbiota remodeling and reduced trimethylamine-N-oxide synthesis, while also reducing neuroinflammation and delaying plaque and amyloid pathology in Alzheimer’s disease models [74,93]. However, resveratrol has also produced null or negative effects, depending mainly on dose and health status. In non-obese women with normal glucose tolerance, 75 mg/day for 12 weeks did not improve insulin sensitivity, body composition, plasma lipids, resting metabolic rate, AMPK, or SIRT1 [85]. Additionally, a very high dose of 3000 mg/day in men with non-alcoholic fatty liver disease did not reduce insulin resistance or hepatic steatosis and significantly increased ALT and AST, suggesting liver stress [91]. Although moderate intake appears safer, high doses or long-term use have been associated with severe gastrointestinal distress, febrile leukopenia, thrombocytopenia, and hormonal or thyroid dysregulation [14,89].

These differences in outcomes suggest that polyphenol efficacy depends on several factors. First, polyphenols appear to work better in metabolically compromised individuals and those at higher cardiovascular risk, while healthy and lean subjects with normal glucose tolerance may show little or no measurable benefit [85,87]. Furthermore, resveratrol and other polyphenols may follow a hormetic pattern, meaning that low-to-moderate doses can be protective, whereas high pharmacological doses may be ineffective or harmful [14,91]. Bioavailability is another major limitation because polyphenols are rapidly metabolized and conjugated into sulfates and glucuronides in the intestine and liver, leaving little free parent compound in circulation [4,79].

Additionally, gut microbiota strongly affects individual responses. Polyphenols are metabolized by gut bacteria into smaller, absorbable, bioactive metabolites, but microbiome composition varies widely between individuals, which influences bioavailability and biological effects [63,94]. The form of intake also matters. Isolated extracts may act differently from whole foods because they can lack fermentable fibers, which normally work together with polyphenols to reduce cholesterol and inflammation [36]. Finally, some bioactive compounds may have stronger effects when combined, such as epigallocatechin-3-gallate from green tea with caffeine or other alkaloids, which can increase overall therapeutic efficacy compared with isolated compounds alone [17].

Overall, the evidence indicates that resveratrol and related polyphenols may provide metabolic, cardiovascular, and neuroprotective benefits, mainly in individuals with metabolic dysfunction or elevated risk. However, their effects are inconsistent because they depend on dose, health status, bioavailability, gut microbiota, food matrix, and compound combinations. Moderate doses may be beneficial, while high doses can be ineffective or harmful.

### 3.4. Green Tea Polyphenols and EGCG-Based Interventions

Green Tea. Green tea (moderately effective) is a widely consumed, nonfermented beverage derived from the leaves of the Camellia sinensis plant [50]. Its health-promoting properties are primarily attributed to its high concentration of green tea polyphenols (GTP), particularly catechins, a subclass of flavonoids [50]. Among these, epigallocatechin-3-gallate (EGCG) is the most abundant and pharmacologically active catechin, with potent antioxidant, anti-inflammatory, antibacterial, antiangiogenic, and neuroprotective effects [50]. Green tea and its extracted polyphenols are also widely recognized for their anti-obesity properties [54]. Clinical interventions show that green tea catechin consumption can significantly reduce body weight, body mass index (BMI), waist circumference, and both total abdominal and visceral fat [44,63]. These metabolic benefits appear to be mediated through several physiological mechanisms, including the inhibition of gastrointestinal enzymes involved in lipid and sugar absorption, the reduction in uric acid through xanthine oxidase inhibition, the suppression of leptin expression in adipose tissue, and the activation of AMPK by EGCG to promote fat decomposition [49,50]. In addition, administration of 400 mg/day for 12 weeks resulted in a significant decrease in uric acid concentration, reduced body fat percentage, decreased ovarian volumes, and delayed early sexual development in obese girls [49,50].

Epigallocatechin Gallate (EGCG) (low–moderate efficacy). Epigallocatechin gallate (EGCG) is the most abundant and pharmacologically active catechin polyphenol found in green tea, and it has been widely studied for its potent biological properties, including its ability to combat obesity, modulate metabolism, and reduce inflammation [50,54]. EGCG exerts its biological and anti-obesity effects through several mechanisms [50]. It inhibits the action of the enzyme catechol-O-methyl-transferase, thereby prolonging the action of catecholamines, which are critical for increasing intracellular cyclic AMP and thus stimulating thermogenesis and fat oxidation [95]. Furthermore, EGCG can inhibit gastrointestinal enzymes involved in lipid and carbohydrate absorption and activate AMP-activated protein kinase (AMPK) to promote fat breakdown and inhibit fat formation [50]. EGCG is also known to exert physiological effects via the 67 kDa laminin receptor (67LR), and its efficacy may be reduced when expression of this receptor decreases [54].

EGCG Combined with α-Glucosyl Hesperidin (moderately effective). Hesperidin is a flavonoid, specifically a flavanone, and a well-known citrus polyphenol that provides a range of physiological benefits, including antioxidant, anti-inflammatory, and hypertension-inhibitory effects [54]. A highly water-soluble derivative of this compound, α-glucosyl hesperidin, is frequently used as a food additive [54]. After consumption, α-glucosyl hesperidin is converted into hesperetin in the gut and can then be further metabolized into eriodictyol in the liver and kidneys; accordingly, its physiological benefits in vivo are largely attributed to the actions of hesperetin and eriodictyol [54]. Daily administration of 146 mg of EGCG and 178 mg of α-glucosyl hesperidin for 12 weeks prevented weight gain and reduced BMI, particularly in subjects under 50 years old, and α-glucosyl hesperidin appears to enhance the functionality of EGCG through its conversion into hesperetin and eriodictyol, with age-related differences likely linked to laminin receptor expression [54].

EGCG Combined with Resveratrol (low–moderate efficacy). The administration of 282 mg/d of EGCG and 80 mg/d of resveratrol for 12 weeks resulted in the downregulation of gene pathways related to adipogenesis, cell cycle regulation, oxidative stress, and inflammation, thereby lowering the energy requirements of adipose tissue. Additionally, although no overall changes in gut microbiota composition were observed, baseline microbiota successfully predicted improvements in skeletal muscle mitochondrial oxidative capacity in men, likely due to their lower baseline abundance of short-chain fatty acid-producing bacteria [63]. At the same time, the administration of 282 mg/day of EGCG and 200 mg/day of resveratrol for 3 days increased resting and postprandial energy expenditure and improved metabolic flexibility in men, likely reflecting a synergistic enhancement of circulating leptin concentrations and the induction of a more oxidative phenotype in skeletal muscle [64].

### 3.5. Curcumin

Curcumin (highly effective) is an active polyphenolic compound derived from turmeric (Curcuma longa) and has demonstrated significant therapeutic potential in the management of conditions such as type 2 diabetes mellitus [23,32]. Curcumin treatment improves the overall function of insulin-producing β-cells and significantly reduces insulin resistance [23]. It also favorably modulates adipocytokines, which are proteins secreted by adipose tissue, by significantly increasing anti-inflammatory adiponectin levels and reducing leptin levels, a hormone that plays a vital role in body weight regulation [23]. Furthermore, advanced delivery forms such as nano-curcumin have been shown to reduce fasting blood glucose and HbA1c levels in patients with diabetes [23]. The administration of 1500 mg/day of curcumin for 12 months resulted in significantly decreased fasting blood glucose, lower glycated hemoglobin, and reduced body weight. It also improved β-cell function, decreased insulin resistance, elevated adiponectin, and lowered leptin. Curcumin’s anti-inflammatory properties help maintain healthy β-cells and reduce overall insulin resistance [23].

### 3.6. Coffee and Caffeine

Coffee. Coffee (moderately effective) is a widely consumed beverage and a major dietary source of polyphenols, particularly hydroxycinnamic acids such as chlorogenic acid, as well as caffeine, lignans, and potassium [43,44]. A substantial body of evidence indicates that regular coffee consumption, especially at an intake of three to five cups per day, confers multiple health benefits, including a reduced risk of heart disease and type 2 diabetes, lower levels of inflammation, and improved mental well-being [44]. Moreover, the administration of three cups per day for 12 weeks resulted in a significant reduction in fat mass and body fat percentage, together with an increase in skeletal muscle mass. These effects may be explained by the action of phenolic compounds, particularly chlorogenic acid, which may inhibit lipogenesis and upregulate AMPK, as well as by caffeine, which increases energy expenditure and thermogenesis [43].

Caffeine. Caffeine (1,3,7-trimethylxanthine) (moderately effective) is a prominent bioactive compound naturally found in coffee, tea, guarana, and various dietary supplements [43,95]. It has been widely studied for its effects on weight management, energy metabolism, and cardiovascular health, and it may also act synergistically with plant polyphenols such as catechins and hydroxycinnamic acids [43,44]. Mechanistically, caffeine influences energy homeostasis by inhibiting the phosphodiesterase-mediated degradation of intracellular cyclic adenosine monophosphate (cAMP), which increases norepinephrine release and supports catecholamine-driven thermogenesis [44]. As a result, it promotes thermogenesis, fat oxidation, and daily energy expenditure [44]. Furthermore, the administration of 160 mg of caffeine daily through two cups of coffee for 6 months resulted in a significant reduction in BMI and body fat percentage compared with green tea and placebo, likely due to its effects on cAMP, norepinephrine release, thermogenesis, and fat oxidation [45].

### 3.7. Hibiscus-Based Extracts and Polyphenol Combinations

*Hibiscus sabdariffa*. *Hibiscus sabdariffa*, a member of the *Malvaceae* family, also known as roselle, contains organic acids and numerous polyphenolic compounds [66,88]. Its consumption exerts favorable effects on metabolic parameters, as these polyphenolic extracts inhibit hyperglycemia, hyperlipidemia, and glycation-oxidative stress while improving insulin resistance [88]. In addition, administration of two capsules three times daily for 12 weeks (moderately effective) resulted in reductions in body weight, BMI, body fat, waist-to-hip ratio, and free fatty acids, together with improvement in liver steatosis. These effects are likely related to the action of polyphenols, which inhibit hepatic lipogenesis and promote hepatic lipid clearance through downregulation of PPAR-γ and SREBP-1c [45]. Administration of 1000 mg/day of *Hibiscus sabdariffa* L. aqueous calyx extract for 12 weeks (null/negative efficacy) also significantly reduced serum LDL levels. However, it did not significantly improve insulin resistance, BMI, waist circumference, or blood pressure compared with placebo. These limited overall effects were attributed to high participant diversity, insufficient study duration, suboptimal dosing, and interindividual variability [88].

Hibiscus and Inulin Beverage (low–moderate efficacy). Inulin is a well-established prebiotic classified as an inulin-type fructan, and as a non-digestible dietary component, it is selectively utilized by beneficial gut microorganisms, particularly Bifidobacteria. Additionally, when fermented by the gut microbiota, inulin produces short-chain fatty acids such as acetate and propionate, which stimulate G-protein-coupled receptors including GPR41/43 and are associated with improved insulin sensitivity and enhanced lipid oxidation. Furthermore, administration of a 60 mL shot of a standardized Hibiscus–inulin beverage for 8 weeks resulted in significant reductions in the Atherogenic Index of Plasma (AIP) and the triglyceride-glucose (TyG) index. The proposed mechanisms include enhanced anthocyanin absorption by hibiscus acid, downregulation of lipogenic enzymes by phenolic compounds, and the production of short-chain fatty acids through inulin fermentation [66].

*Lippia citriodora*, *Hibiscus sabdariffa*, and Bergamot Polyphenol Extract (highly effective). Similarly, the administration of 500 mg/day of a polyphenolic extract combination from Lippia citriodora and *Hibiscus sabdariffa* for two months resulted in significant reductions in body weight, abdominal circumference, body fat percentage, heart rate, and systolic blood pressure in overweight subjects. These effects appear to be mediated through modulation of fat metabolism via AMPK activation, with decreased intracellular triglycerides, increased energy expenditure, and restoration of the balance between hunger and energy expenditure through increased GLP-1 and reduced resistin and leptin levels [2,41]. In parallel, the administration of 650 mg or 1300 mg of Bergamot polyphenol extract daily for 90 days resulted in significant reductions in fasting glucose, triglycerides, cholesterol parameters, and body weight. The likely explanation is that pectin enrichment lessens appetite, while modulation of circulating hormones, including reduction in leptin and ghrelin and upregulation of adiponectin, helps balance caloric intake [33].

### 3.8. Fruits, Berries, and Polyphenol-Rich Plant Foods

This section reviews clinical evidence from studies investigating polyphenol-rich fruits, berries, and other plant-derived foods, including fruit extracts, grape-derived products, and flavonoid-rich vegetables, all of which share a common capacity to modulate adiposity and cardiometabolic risk through overlapping polyphenol-mediated mechanisms. Interventions are organized beginning with combination extracts and whole dietary patterns, followed by individual fruits and berries, and concluding with grape-derived fermented products.

*Litchi chinensis* and Tea Extract Combination (moderately effective). Another polyphenol combination with significant results is a formulation derived from Litchi chinensis (lychee) fruit extract and tea extracts, which, when administered daily at 200 mg for 12 weeks, significantly reduced abdominal visceral fat area by enhancing PPAR-α expression to accelerate mitochondrial β-oxidation and decreasing perilipin expression to promote lipolysis [46].

Juçara. Juçara (*Euterpe edulis Martius*) (moderately effective) is a fruit-bearing palm tree native to the Brazilian Atlantic Forest and is frequently referred to as the “açaí from the Atlantic Forest” because of its similarities to açaí [47,48]. However, juçara is considered a “super fruit” because, in some respects, its nutritional value exceeds that of açaí, containing approximately four times higher amounts of certain minerals and anthocyanins [47,48]. The pulp of the juçara fruit is highly nutritious, providing a substantial profile of dietary fibers, proteins, and sugars, together with a distinctive lipid fraction composed of 38% monounsaturated fatty acids (MUFA), 20% polyunsaturated fatty acids (PUFA), and 37.4% saturated fatty acids (SFA) [47,48]. In addition, juçara is exceptionally rich in polyphenols, particularly the anthocyanins cyanidin 3-rutinoside and cyanidin 3-glucoside, which are associated with potent biological activities and health benefits [47,48]. Administration of 5 g for 6 weeks resulted in reduced body fat, increased HDL-c, doubled serum adiponectin levels, increased fecal acetate excretion, and a rise in the abundance of *A. muciniphila*, *Bifidobacterium* spp., and *C. coccoides*. These effects are likely explained by the action of polyphenols and fatty acids in modulating intracellular signaling to enhance anti-inflammatory responses, while the berry’s composition also appears to promote a bifidogenic effect that mediates acetate production [47,48].

Persimmon and Karonda Microencapsulated Formulation (moderately effective). A microencapsulated polyphenol formulation made from persimmon (*Diospyros kaki* L.) and karonda (*Carissa carandas* L.) is presented as a promising strategy for improving metabolic health in individuals who are overweight or have prediabetes [52]. The formulation is prepared using a 70% persimmon and 30% karonda ratio and contains several important polyphenols, including quercetin, ferulic acid, chlorogenic acid, and cyanidin-3-glucoside [52]. Additionally, cyanidin-3-glucoside is highlighted for its strong antioxidant and anti-inflammatory activities [52]. Microencapsulation also improves the stability and systemic bioavailability of these compounds compared with non-encapsulated extracts, which have poor absorption. Moreover, administration of 162 mg/day for 8 weeks resulted in improved glycemic control, reduced systemic inflammation, increased adiponectin, improved lipid profiles, and enhanced physical fitness. Physical exercise activates AMPK, while polyphenols strengthen this signaling and inhibit proinflammatory cascades [52].

Apples. Apples are among the most widely consumed fruits worldwide and represent an important dietary source of fiber, micronutrients, and phenolic compounds [34]. They are especially rich in soluble fiber, particularly pectin, as well as polyphenols such as flavanols, procyanidins, and hydroxycinnamic acids, which are found in particularly high concentrations in the peel [34,96]. Evidence suggests that apple consumption confers several important health benefits, particularly in relation to inflammation, body fat regulation, and metabolic health [9,34,96]. In one study (moderately effective), administration of 750 mL/day of apple juice for 4 weeks significantly reduced percent body fat and increased lean body mass, without affecting BMI or plasma lipid levels. Notably, the reduction in body fat was observed exclusively in carriers of the IL-6-174 C/C genotype, indicating that apple polyphenols may regulate lipid metabolism in a manner influenced by genetic background [9]. Consumption of 3 whole Gala apples per day for 6 weeks reduced fasting plasma *C-*reactive protein, IL-6, and LPS-binding protein, and also decreased PBMC-secreted IL-6 and IL-17; these effects are likely related to the ability of apple polyphenols to exert antioxidative effects and modulate cell signaling cascades involved in inflammatory cytokine production (low efficacy) [34]. However, during an acute 6 h postprandial period, apple intake did not alter postprandial triacylglycerol responses and instead resulted in exaggerated insulin secretion, likely due to the presence of digestible carbohydrates such as fructose and sucrose [96].

Camu-camu (moderately effective). Camu-camu (CC) is an Amazonian fruit rich in polyanthocyanidins (PAC) and ellagitannins, and it has strong antioxidant and anti-inflammatory activity [55]. Recent research suggests that it may benefit liver health, modulate gut microbiota, and improve metabolic conditions [55]. Additionally, supplementation with 1.5 g/day for 12 weeks reduced hepatic steatosis by 15.85% and lowered plasma AST and ALT levels compared with placebo. These effects may be related to the prebiotic-like action of ellagitannins and proanthocyanidins on gut microbiota composition [55].

Grapes, Grape Pomace, and Pomegranate. Grapes and their derivatives, such as freeze-dried grape powder, grape pomace, and red wine extracts, are rich in phenolic compounds, including flavonoids such as anthocyanins and flavanols, phenolic acids, and stilbenes such as resveratrol. Owing to this robust phytochemical profile, grapes have been widely investigated for their potential to improve cardiovascular and metabolic health. Consuming grapes alongside a meal may help mitigate some of the metabolic stress induced by poor diets [32,83,86].

In obese individuals, consumption of 60 g of freeze-dried, polyphenol-rich whole grape powder, equivalent to 2.2 cups of fresh, commercially available grapes, alongside a high-fat, high-carbohydrate meal produced acute protective cardiovascular effects. Specifically, grape powder significantly decreased plasma concentrations of endothelin-1, a potent vasoconstrictor, 5 h after the meal and increased expression of NRF2, a transcription factor involved in oxidative stress defense, in immune cells 3 h post-meal. In addition, previous research has shown that acute supplementation with grape pomace extract alongside a standard meal can improve postprandial glucose and triglyceride levels in healthy individuals [32,86].

Despite these acute postprandial effects, daily grape consumption over a longer period (null/negative efficacy) has yielded mixed results for fasting biomarkers. Four weeks of daily whole grape powder consumption did not produce clinically significant changes in fasting inflammatory biomarkers or endothelial function in obese participants. Chronic intake did, however, increase the overall concentration of phenolic metabolites in the blood, primarily through byproducts of colonic bacterial metabolism [32]. Notably, the same four-week intervention also produced an unexpected increase in soluble vascular cell adhesion molecule 1 (sVCAM-1) concentrations, while other markers of inflammation remained unchanged, an effect potentially influenced by baseline adiposity and pharmacokinetic habituation [32].

Grape pomace, a byproduct of winemaking, contains high levels of both extractable and non-extractable polyphenols, including proanthocyanidins and malvidin 3-O-glucoside. Although 6 weeks of chronic supplementation with whole grape pomace has previously been shown to improve fasting glucose and fasting insulin in subjects with cardiometabolic risk, acute interventions have been less successful [86].

A clinical trial using a single 10 g dose of a mixed grape and pomegranate pomace supplement (null/negative efficacy) did not significantly improve glucose metabolism, insulin response, or plasma and urine antioxidant capacity during a standard oral glucose tolerance test in adults with abdominal obesity, likely because this nutritional dose was not sufficient to significantly increase circulating polyphenols at the selected sampling times. However, when the supplement was consumed 10 h before the glucose challenge, there was a non-significant tendency toward improved fasting insulin sensitivity [86]. Pomegranate is also a rich source of polyphenols, particularly anthocyanins and ellagitannins, which are polymeric structures containing gallic acid and ellagic acid units bound to sugar moieties. Research on pomegranate juice and extracts has shown diverse effects on the microbiome, inflammation, and cardiometabolic health, and pomegranate extract consumption has been reported to induce the formation of ellagitannin metabolites and favorably alter stool microbiota in healthy volunteers [55,86].

Cherries (low–moderate efficacy). Cherries, including varieties such as dark sweet cherries (*Prunus avium*), Bing sweet cherries, and tart cherries, are fiber-rich fruits that also contain bioactive compounds such as polyphenols [68]. They are an important source of phenolic acids and anthocyanins, the latter of which are responsible for their characteristic deep red color. These nutritional components have been associated with multiple health benefits, particularly in reducing risk factors linked to obesity and cardiometabolic diseases. Administration of 400 mL cherry juice per day for 30 days resulted in reductions in both systolic and diastolic blood pressure, as well as decreased levels of the pro-inflammatory cytokine interferon-gamma. These effects are likely attributable to the antioxidant activity of polyphenols, which inhibit reactive oxygen species and suppress pro-inflammatory signaling pathways [68].

Blackberries (low–moderate efficacy). Blackberries have been studied for their potential to improve cardiometabolic health in overweight and obese individuals. In this context, consuming 600 g of whole blackberries per day, equivalent to about 4 cups, for seven days increased fat oxidation in overweight and obese men [61]. This effect was shown by a reduction in the 24 h respiratory quotient, which indicated a greater use of fat rather than carbohydrate for energy, and overall fat oxidation increased by 7% compared with a carbohydrate- and calorie-matched control diet. Moreover, this increase in fat oxidation was maintained throughout the day, including at night, after meals, and during 30 min of low-intensity treadmill walking. Blackberry consumption also improved glucose metabolism and insulin sensitivity, as participants showed a lower insulin response during meal-based glucose tolerance tests while maintaining blood glucose levels similar to those of the control group [61]. Additionally, HOMA-IR improved, indicating better insulin sensitivity, and this effect appeared to be age-dependent, with younger subjects showing the greatest response to the intervention. Overall, the administration of 600 g/day for seven days increased fat oxidation and improved insulin sensitivity, with proposed mechanisms including potential AMPK activation in tissues involved in glucoregulation, reduced fatty acid overload through a beige-adipocyte phenotype, and reduced glucose absorption [61].

Elderberries (low–moderate efficacy). Elderberries are a remarkably dense source of polyphenols, particularly cyanidin-based anthocyanins, which have been extensively studied for their potential to counteract obesity and its associated metabolic complications, including diabetes, dyslipidemia, and cardiovascular disease [62]. Recent clinical and preclinical studies have highlighted several important health benefits associated with elderberry consumption. In a clinical trial involving overweight and obese adults, the daily consumption of 355 g of 100% elderberry juice, providing approximately 720 mg of cyanidin-based anthocyanins, for one week significantly improved whole-body energetics. Following a high-carbohydrate meal challenge, elderberry juice reduced the blood glucose response curve by 24% and the insulin response curve by 9%. The administration of 355 g per day for one week also resulted in significant increases in Firmicutes and Actinobacteria populations, a 24% reduction in postprandial blood glucose response, and increased fat oxidation. High concentrations of cyanidin-based anthocyanins appear to modulate the gut microbiota, thereby mediating improvements in glucose regulation [62].

Strawberries and Strawberry–Cranberry Polyphenol Extracts. Dietary strawberries and their polyphenol extracts provide significant health benefits, particularly in the management of osteoarthritis, the improvement of insulin sensitivity, and the enhancement of cardiovascular health [32,36]. Strawberries contain bioactive compounds that improve glucoregulation and help combat insulin resistance. A 6-week clinical trial demonstrated that supplementation with a beverage containing 333 mg of strawberry and cranberry polyphenols (SCP) significantly improved whole-body insulin sensitivity in overweight and obese, insulin-resistant adults. Furthermore, the SCP beverage reduced first-phase insulin secretion, measured by *C-*peptide levels, during an oral glucose tolerance test, suggesting a protective effect against pancreatic β-cell exhaustion (low–moderate efficacy) [36]. In addition, administration of 50 g of freeze-dried strawberry powder daily for 12 weeks significantly reduced constant, intermittent, and total knee pain scores, while also lowering markers of disability (low efficacy) [32]. It also decreased serum biomarkers of inflammation and cartilage degradation, including MMP-3, likely because the naturally rich antioxidant polyphenols help counteract inflammation [32].

Similarly, a mixture prepared from dried strawberry (Fragaria × ananassa Duch.) and cranberry (*Vaccinium macrocarpon* L.) polyphenol extracts contains approximately 18% total polyphenols [36]. Its principal constituents are proanthocyanidins and phenolic acids, particularly p-coumaric acid, m-coumaric acid, and coumaroyl glucoside, which are absorbed and increase circulating plasma metabolites after consumption. In a 6-week, double-blind, randomized trial, overweight or obese insulin-resistant adults consumed 1.84 g/day of this strawberry–cranberry extract mixture, providing 333 mg of polyphenols; this dose was selected on the basis of prior rat bioavailability studies. This intervention improved insulin sensitivity and prevented a further compensatory increase in early-phase insulin secretion, likely through enhancement of insulin signaling and glucose transport in skeletal muscle cells mediated by circulating metabolites such as p-coumaric acid [36].

Blueberries. Blueberries are a low-calorie functional food rich in polyphenols, anthocyanins, phenolic acids, fiber, and essential micronutrients, with antioxidant and anti-inflammatory effects across multiple physiological systems [65,84]. They improve glucose tolerance and insulin sensitivity, partly by inhibiting starch-digesting enzymes and activating AMPK, which supports glucose uptake and reduces insulin resistance [36,75]. When combined with soluble fiber, blueberry supplementation can also reduce gestational weight gain and gestational diabetes risk in high-risk pregnant women with obesity [65]. In addition, blueberry polyphenols act as prebiotics, promoting beneficial gut bacteria and improving polyphenol metabolism [62,65,84]. Soluble fibers such as pectin and oat beta-glucan increase gastrointestinal viscosity, delay gastric emptying, promote satiety, and support body weight regulation, while their fermentation produces short-chain fatty acids that help regulate appetite, energy homeostasis, and glucose and lipid metabolism [33,75,96] (low–moderate efficacy). Consistent with these effects, daily intake of 280 g of whole blueberries and 12 g of soluble fiber for 18 weeks lowered maternal weight gain, *C-*reactive protein, and postprandial blood glucose [65].

Administration of 36 g/day of lyophilized blueberry powder for 12 weeks (null/negative efficacy) significantly enriched *Coriobacteriales incertae* sedis in the fecal microbiome and also reduced circulating total HDL-P and ApoA-I. This increase likely reflects a compensatory adaptation that supports the digestion, absorption, and metabolism of greater amounts of dietary polyphenols [84].

Mulberries (low–moderate efficacy). Mulberries (*Morus* spp.) are widely cultivated fruits packed with phytochemicals such as polyphenols, including anthocyanins, flavonoids, and phenolic acids, that have traditionally been used to manage diabetes, reduce cholesterol, combat obesity, and provide antioxidant benefits [69]. The three primary species of mulberry are each characterized by distinct phytochemical profiles and health applications. Red mulberries (*Morus rubra*) are rich in anthocyanins and exhibit strong antioxidant and anti-inflammatory properties; black mulberries (*Morus nigra*) contain high levels of resveratrol, which is recognized for its neuroprotective and anti-cancer effects, and white mulberries (Morus alba) are abundant in flavonoids and alkaloids, which are primarily known for their hypoglycemic and lipid-lowering activities. The clinical efficacy of these fruits is evident in findings where the administration of 100 g daily for 6 weeks resulted in significantly reduced systolic and diastolic blood pressure, mean arterial pressure, triglycerides, and *C-*reactive protein levels. Ultimately, these bioactive compounds (anthocyanins and polyphenols) exert strong antioxidant and anti-inflammatory effects, improving endothelial function and increasing nitric oxide bioavailability [69].

Cranberries (null/negative efficacy). Cranberries are a rich source of dietary polyphenols, including anthocyanins, proanthocyanidins (PACs), flavonols, and phenolic acids, and these compounds are associated with high antioxidant capacity and several cardiometabolic and health benefits [36,82]. Moreover, cranberry research has mainly focused on insulin sensitivity, cardiovascular risk factors, oxidative stress, and the gut microbiome [47,82]. Findings on insulin sensitivity and glucose metabolism have been mixed, depending on the form of cranberry used and whether it was combined with other components. Consuming 450 mL cranberry juice daily for 8 weeks did not significantly change insulin sensitivity, although it did reduce triglycerides and oxidative stress markers in participants with high baseline *C-*reactive protein levels [36,82].

Chokeberry (null/negative efficacy). *Aronia melanocarpa*, or chokeberry, is rich in polyphenols, especially anthocyanins, and has been studied for its potential cardiovascular and metabolic benefits [87]. Some studies suggest that chokeberry may reduce total cholesterol, LDL cholesterol, and blood pressure. However, the evidence is inconsistent. In a four-week placebo-controlled trial, chokeberry juice did not significantly improve total cholesterol, LDL cholesterol, or blood pressure compared with placebo. Additionally, it changed plasma phospholipid fatty acids by increasing palmitic acid and decreasing linoleic acid, suggesting an effect on fatty acid absorption and elimination. Moreover, the observed blood pressure reduction was likely due to a placebo effect rather than the chokeberry intervention itself [87].

Whole fruits are beneficial, yet consuming large amounts of fruit juice is flagged as a potential risk factor because of its high sugar content [34]. Drinking more than 150 mL of fruit juice per day over an extended period has been linked to body weight gain [34]. Similarly, high intakes of fruit juice among pregnant women have been associated with increased risks of hyperglycemia, excess weight gain, and gestational diabetes mellitus [24]. For this reason, whole fruit is considered the healthier option, since the dietary fiber in the skin and pulp helps offset the sugar content [96].

Beyond effects on body weight, the high fructose content in fruit juices can actively counteract the cardioprotective effects of polyphenols [70]. In a 4-week trial of cloudy apple juice, researchers observed an increase in fasting triglyceride levels, which they attributed to the fructose intake provided by the juice (about 50.3 g/day) [70]. This effect occurs because fructose stimulates hepatic triglyceride synthesis and decreases triglyceride clearance [70].

Natural sugars can mask the benefits of bioactive compounds even in whole fruits [96]. In an acute meal trial of whole Gala apples, researchers found no improvement in postprandial lipemia, noting that the apples provided about 20.8 g of digestible carbohydrates, primarily fructose (11.9 g) [96]. Fructose also promotes postprandial hypertriacylglycerolemia by lowering the activation of adipose tissue lipoprotein lipase, increasing de novo lipogenesis, and promoting greater enterocyte chylomicron secretion [96]. Consequently, the authors hypothesized that this fructose-induced lipid spike may have completely counteracted any lipid-lowering potential from the apples’ pectin and polyphenols [34,96].

To avoid these confounding effects of natural sugars, some clinical trials use specific extraction or formulation methods [52]. For example, in a study testing a concentrated Thai mulberry drink, researchers used maltitol, a sugar alcohol, to replace sucrose [23]. This produced a low-glycemic beverage with negligible natural sugars, making it suitable for assessing cardiovascular benefits in patients with metabolic restrictions [52].

Onion Peel, Quercetin, and Rutin (moderately effective). Onion peel extract is a rich source of quercetin, a potent antioxidant flavonoid associated with improvements in inflammatory responses and endothelial dysfunction [53]. Quercetin is a polyphenol widely found in fruits and vegetables, with particularly high concentrations in onions, and it is also present in cranberries, grapes, persimmons, and karonda [32,52,53,82]. Rutin, additionally, is a prebiotic flavonoid and the glycoside form of quercetin [31]. Unlike quercetin, which is absorbed mainly in the small intestine, rutin is absorbed more slowly after bacterial fermentation in the colon (null/negative efficacy) [31]. Moreover, daily administration of 100 mg of quercetin for 12 weeks improved flow-mediated dilation and increased circulating endothelial progenitor cells, likely because it reduces oxidative stress and inflammation through its antioxidant action [53]. Rutin has also shown promising anti-diabetic properties in animal and in vitro models, including prevention of human amylin aggregation into toxic pancreatic amyloid deposits that impair insulin secretion and damage pancreatic β-cells [31]. However, in humans, 500 mg/day of rutin for 12 weeks had no effect on pancreatic beta-cell function or gut bacterial community composition [31].

Red Wine. Red wine is a major source of dietary polyphenols, including resveratrol, flavanols, and anthocyanins, and it is a key component of the Mediterranean dietary pattern, which is associated with better health-related quality of life and a lower risk of chronic diseases [30,97]. Moreover, research suggests that red wine polyphenols may provide important health benefits, particularly in cardiovascular health, metabolic syndrome, and gut microbiota modulation. Additionally, one of the main mechanisms involved is the prebiotic effect of red wine on the gut microbiome. Administration of 272 mL per day for 30 days (moderate efficacy) increased intestinal barrier-protecting and butyrate-producing bacteria and improved metabolic syndrome markers. Furthermore, these effects were linked to polyphenol-induced changes in the gut microbiota, which enhanced intestinal barrier integrity and reduced inflammatory lipopolysaccharides [58].

Red Wine Polyphenol Extract: Limited Effects in Obese Subjects (null/negative efficacy). However, red wine polyphenol extract was evaluated in a randomized, double-blind, placebo-controlled trial investigating whether red wine polyphenols could improve obesity-associated insulin resistance [83]. In this study, obese participants received a high dose of 600 mg/day of red wine polyphenol extract for 8 weeks, administered as two 300 mg scarlet gelatin capsules, one taken with breakfast and one with dinner. However, supplementation with 600 mg/day of red wine polyphenols for 8 weeks did not produce significant changes in insulin sensitivity, glucose regulation, or lipid levels in these obese volunteers, possibly because the participants were metabolically too healthy to exhibit a measurable response or because dietary proteins may have affected polyphenol absorption [83].

### 3.9. Whole Grains, Grain-Derived Products, and Nuts

Rice Bran Oil (moderately effective). Rice bran oil (RBO) is a heart-friendly byproduct of the rice-milling process, highly valued for its dense phytochemical profile and therapeutic potential in metabolic disorders [56]. It is composed of approximately 48.9% monounsaturated fatty acids, primarily oleic acid, and 32.5% polyunsaturated fatty acids, primarily linoleic acid. Its most potent bioactive components include γ-oryzanol, γ-tocotrienol, and α-tocopherol. Administration of 30 g/day for 8 weeks significantly lowered total cholesterol, LDL-cholesterol, fasting blood glucose, malondialdehyde, and insulin resistance markers. These effects are likely mediated by γ-oryzanol, which suppresses hepatic cholesterol synthesis, as well as by the oil’s unsaturated fatty acids and bioactive compounds, which inhibit intestinal glucose-regulating enzymes [56].

Pigmented Rice (low efficacy). Pigmented rice varieties, particularly red and purple rice, are considered functional foods because they contain high levels of polyphenols, especially anthocyanins such as cyanidin-3-glucoside and peonidin-3-glucoside [77]. Moreover, these compounds have strong free-radical scavenging and metal-chelating properties. Additionally, an acute dietary intervention with one cup of cooked pigmented rice significantly increased plasma antioxidant activity and reduced biomarkers of lipid peroxidation and pro-inflammatory cytokines in obese individuals. Furthermore, the bioavailability of anthocyanins and phenolic acids supports therapeutic pathways involved in scavenging free radicals and modulating inflammation [77].

Whole-Grain Wheat (low efficacy). Whole-grain (WG) wheat is a rich source of dietary fiber and phenolic compounds, which contribute substantially to health benefits, including a reduced risk of chronic diseases such as cardiovascular disease, diabetes, and cancer [76]. WG wheat is particularly characterized by a high content of hydroxycinnamic acids, of which ferulic acid (FA) is the most abundant. Its nutritional density is markedly greater than that of refined wheat; for example, a 70 g daily portion of a 100% WG wheat product provides 138.7 mg of total phenolics and 96.7 mg of FA, whereas an equivalent portion of refined wheat provides only 2.6 mg of phenolics and 2.6 mg of FA. Approximately 95% of these phenolic compounds are covalently bound to arabinoxylan chains within the cell wall polysaccharides of the cereal dietary fiber. The administration of 70 g/d of a whole-grain wheat product for 8 weeks resulted in elevated serum dihydroferulic acid, increased excreted ferulic acid, reduced plasma tumor necrosis factor-α, and shifts in fecal microbiota composition. These effects are likely explained by bacterial fermentation of polysaccharides, which releases ferulic acid and is directly associated with the observed improvement in inflammatory immune response [76].

Whole Pecans (low–moderate efficacy). Whole pecans are tree nuts with a rich nutritional profile, providing a good source of monounsaturated fats, such as oleic acid, polyunsaturated fats, such as linoleic acid, and fiber [35]. They also contain a wide range of phytochemicals, including total phenols, proanthocyanidins, hydrolysable tannins, and flavonoids, along with fat-soluble bioactives such as phytosterols and tocols, and essential minerals including magnesium, manganese, and zinc. The administration of approximately 42.5 g of whole pecans daily for 4 weeks resulted in significant improvements in serum insulin, insulin resistance (HOMA-IR), and beta cell function (HOMA-B). These effects may be explained by the increased monounsaturated fatty acids, which likely enhanced cell membrane fluidity and insulin responsiveness, while pecan polyphenols may have helped protect pancreatic beta cells [35].

Pelemir (low efficacy). Pelemir (*Cephalaria syriaca*) is a bitter-tasting, cold-resistant, and drought-tolerant functional seed crop that has traditionally been used in Anatolia to improve dough rheology and extend the shelf life of bread [78]. Although it was previously misclassified as a weed, pelemir is now emerging as a functional food ingredient with notable nutritional and pharmacological potential. Its seeds contain 9–30% dietary fiber, 14–21% protein, and polyunsaturated fats such as linoleic and oleic acids. In addition, the plant is rich in bioactive compounds, including polyphenols, triterpenes, and flavonoids, which are associated with antioxidant, anti-inflammatory, and antidiabetic properties. In a postprandial test meal containing 50 g of carbohydrates and 0.3% pelemir flour, administration over 120 min resulted in significantly higher incremental areas under the curve for insulin, *C-*peptide, and GLP-1, without altering glucose levels, suggesting that its bioactive compounds may influence glycemic regulation through non-nutrient-mediated mechanisms such as gut-derived signaling [78].

### 3.10. Spices and Other Bioactive Foods

Cinnamon (moderately effective). Cinnamon, specifically the dried inner bark of trees from the Cinnamomum genus, is a widely available and low-cost spice rich in polyphenols such as cinnamaldehyde, proanthocyanidins, coumarin, catechins, trans-cinnamic acid, and flavones [57]. Because of its diverse bioactive profile, cinnamon has been studied as a potential dietary intervention for improving metabolic health, particularly in the management of blood glucose and lipid levels. Studies suggest that cinnamon can also play a beneficial role in glucose homeostasis, although its effects may vary depending on the species and dosage used. Administration of 4 g/d for 4 weeks lowered continuously monitored 24 h glucose concentrations and reduced glucose peaks, while cinnamon polyphenols, including cinnamaldehyde, may improve insulin sensitivity by activating insulin receptors; additionally, increased glucose-dependent insulinotropic polypeptide may stimulate lipoprotein lipase activity [57].

Licorice Flavonoid Oil (low–moderate efficacy). Licorice is widely consumed as both a food and a natural medicine in Eastern and Western countries, and several species, including *Glycyrrhiza uralensis*, *G. glabra*, and *G. inflata*, contain beneficial flavonoids [72]. Recent research has focused on Licorice Flavonoid Oil (LFO), a functional food ingredient composed of hydrophobic licorice polyphenols primarily extracted from *G. glabra* roots and suspended in medium-chain triglycerides, with glabridin identified as its major active component. In acute double-blind, placebo-controlled crossover studies, administration of LFO at doses of 300–600 mg before light exercise consistently decreased the respiratory exchange ratio and increased fat oxidation in healthy overweight women, effects attributed to suppression of fatty acid synthesis and activation of peroxisomal β-oxidation [72]. These findings suggest a potential role in enhancing lipid metabolism during physical activity, though long-term clinical evidence remains limited.

Tomato Extract (low efficacy). Tomato extract represents another compound with moderate effects on obesity. When administered daily in two divided doses totaling 300 mg over a period of 4 weeks, it resulted in reduced fasting plasma and urinary trimethylamine-N-oxide (TMAO) levels, along with lower plasma lipopolysaccharide (LPS) concentrations. This effect is attributed to modulation of the gut microbiota, particularly through a reduction in bacterial taxa involved in trimethylamine (TMA) production [74].

Melinjo Seed Extract (low efficacy). Melinjo (*Gnetum gnemon* L.) is a gymnosperm native to Indonesia, where its seeds have long been consumed as a traditional food [81]. In recent years, Melinjo Seed Extract (MSE) has attracted scientific interest as a functional food because of its rich phytochemical profile and its promising metabolic health benefits. MSE is notable for containing high levels of polyphenols, particularly trans-resveratrol and its structural dimers and glycosides, including gnetin C, gnetin L, and gnemonosides A, B, C, and D. Although trans-resveratrol is well known for its health benefits, it is limited by low bioavailability and rapid presystemic metabolism. By contrast, gnetin C, which is highly abundant in MSE, shows a much longer blood retention time, averaging 36.3 h compared with only 6.6 h for trans-resveratrol. This pharmacokinetic advantage suggests that MSE may offer more sustained tissue exposure than standard resveratrol supplements, though direct comparative clinical trials are lacking. Administration of 300 mg/day for 14 days increased the ratio of high-molecular-weight to total adiponectin, an effect that appears to occur through the induction of disulfide-bond A oxidoreductase-like protein, which promotes adiponectin multimerization [81].

Rose-hip seeds. Rosehip, defined as the whole fruit of *Rosa canina* L., is an exceptionally nutrient-dense fruit notable for one of the highest vitamin C (ascorbic acid) concentrations found among plants, a property that underlies its value for immune support and scurvy prevention [98,99,100]. Beyond ascorbic acid it supplies vitamin E, carotenoids (precursors of vitamin A), antioxidants, flavonoids, and essential fatty acids, and these constituents are unevenly distributed across the fruit: the seeds contain roughly fourfold higher levels of polyunsaturated fatty acids such as linoleic acid, whereas the fleshy pericarp is enriched in lycopene, vitamin E, galactolipids, and triterpenoids [100].

The principal bioactive responsible for the anti-obesity activity is tiliroside, a glycosidic flavonoid concentrated in the seeds [99,100], which exerts a potent effect on obesity by modifying fat metabolism [98,99]. Consistent with this localization, extracts of the whole fruit or of the seeds specifically reduce body weight and abdominal visceral fat, while extracts derived solely from the pericarp lack such effects owing to the absence of tiliroside [98,100]. At the mechanistic level the seed compounds suppress fat-storage pathways while upregulating the cellular machinery for fat oxidation and energy expenditure [100].

Tiliroside accelerates fat catabolism by promoting lipolysis, the breakdown of stored fat [100]. This action is mediated by increased adiponectin production in adipose tissue and phosphorylation of AMP-activated protein kinase (AMPK) [99,100], which in turn elevates expression of PPAR-α (peroxisome proliferator-activated receptor alpha) in liver and skeletal muscle and thereby stimulates fatty acid oxidation for energy [98,99,100].

In parallel with enhanced lipid breakdown, rosehip seed extracts limit the accumulation of new fat [100]. Their polyphenols act as antagonists of PPAR-γ, the nuclear receptor governing adipogenesis and lipogenesis [99,100], such that downregulation of this lipogenic pathway reduces the differentiation of pre-adipocytes into mature adipocytes and lowers total fat storage [100]. The treatment additionally induces brown adipose tissue (BAT) markers such as uncoupling protein 1 (UCP1) and irisin [100], promoting the conversion of white, fat-storing adipocytes into thermogenically active “beige” cells that expend energy as heat [100]. Finally, rosehip extracts enhance autonomic nervous system activity [99,100]; because visceral fat is highly responsive to the catecholamine-induced lipolysis elicited by this response, the stimulation raises fat utilization both at rest and during exercise and yields targeted reductions in abdominal visceral fat [99].

### 3.11. Dietary Flavonoids, and Multicomponent Polyphenol Interventions

Habitual Dietary Flavonoid Intake (low-moderate efficacy). Flavonoids are a diverse and highly abundant subclass of dietary polyphenols found naturally in a wide variety of plant-based foods, including fruits, vegetables, seeds, tea, and wine [30]. Structurally, they are categorized into several major subclasses, including anthocyanins, which are responsible for the red, blue, and purple pigments in berries, grapes, and dark cherries; flavan-3-ols, such as catechins found in green tea; flavonols, such as quercetin found abundantly in onions and apples; flavanones, such as naringenin, hesperidin, and other citrus polyphenols; and flavones [53]. The habitual dietary intake of high amounts of total flavonoids resulted in a significantly lower likelihood of having excess body weight and obesity. Flavonoids activate catabolic pathways, enhance energy expenditure, and inhibit adipogenesis and lipogenesis by decreasing the expression of lipoprotein lipase, SREBP1c, and PPAR-gamma [30].

Polyphenol-Rich Diet Combined with Marine n-3 PUFA (low–moderate efficacy). The administration of a diet naturally rich in polyphenols, approximately 2800 mg/day, together with marine long-chain n-3 polyunsaturated fatty acids for 8 weeks resulted in reduced fasting and postprandial plasma triglycerides, decreased large VLDLs, lower postprandial chylomicron cholesterol, and a significant reduction in urinary 8-isoprostane. Polyphenols reduce triglyceride absorption or lipogenesis while influencing oxidative stress [67].

Polyphenol Complex (Forskolin, Green Coffee, Green Tea, and Cofactors) (moderately effective). Another mix with promising results in combating obesity consists of forskolin, green coffee, green tea, beet root, α-lipoic acid, vitamin E, and CoQ10, which was administered at a dose of 6 capsules daily for 12 weeks and resulted in significant reductions in body weight, fat mass, and liver enzymes AST and ALT, while fat-free mass was preserved; these effects were associated with improved mitochondrial function and lipid metabolism [42].

## 4. Discussion

### 4.1. Current Pharmacological Standard of Care: Incretin-Based Therapies

The incretin system, mainly composed of GLP-1 and GIP, plays a central role in energy balance and glucose homeostasis, with GLP-1 regulating both blood sugar and appetite [101,102]. GLP-1 supports glucose control by stimulating insulin secretion, suppressing glucagon release, and increasing pancreatic beta-cell sensitivity to glucose, which helps regulate postprandial blood sugar levels [101,102]. Moreover, it reduces appetite and promotes satiety through both central and peripheral mechanisms, as it promotes satiety and reduces cravings in the brain while delaying gastric emptying and slowing food transit in the gastrointestinal tract, thereby prolonging fullness [101,102,103,104]. Additionally, incretins influence fat metabolism and energy expenditure by reducing de novo lipogenesis and lipotoxicity and by activating pathways such as AMPK, SIRT1, and PPARα, which promote fatty acid beta-oxidation, lipolysis, and lipid clearance [105,106]. However, native GLP-1 is rapidly degraded by DPP-4, which led to the development of synthetic GLP-1 receptor agonists such as semaglutide and dual GLP-1/GIP agonists such as tirzepatide [101,102,107].

To contextualize the role of plant-based interventions identified in the preceding results, a brief overview of the current pharmacological standard of care for obesity is provided below.

*Semaglutide* is structurally modified to resist DPP-4 degradation, allowing sustained action in the treatment of obesity and type 2 diabetes [102,107,108,109,110]. It promotes weight loss and metabolic control by reducing appetite and cravings, delaying gastric emptying, increasing insulin secretion, suppressing glucagon release, and improving postprandial glucose regulation [102,103,104,105,106,111]. Semaglutide also improves liver lipid metabolism, activates AMPK and PPAR-alpha, modulates adipose tissue lipid handling, and suppresses inflammatory cytokines such as IL-6, IL-1β, and TNF-alpha [105,106]. Clinical trials demonstrated that subcutaneous semaglutide 2.4 mg produces substantial weight loss across diverse patient populations [8,112]. Adults without diabetes lost 14.9% of their body weight, while those with type 2 diabetes experienced a 9.6% reduction [8,112]. Additionally, combining the medication with intensive behavioral therapy and a low-calorie diet increased adult weight loss to 16.0% [8]. Furthermore, adolescents aged 12 to 18 achieved a 16.1% average weight reduction [113,114].

In this context, obesity should be treated as a chronic disease, as the maintenance of therapeutic benefits depends on ongoing pharmacological intervention, which has been shown to sustain weight loss for up to two years [8,103,105,107,115]. By contrast, discontinuation of treatment leads to rapid weight regain, with patients reportedly regaining approximately two-thirds of the weight lost within one year after therapy cessation [8,11,104,107].

In adults, eligibility for Wegovy (semaglutide) is determined by body mass index (BMI) criteria. Adults with obesity qualify if they present a BMI of 30 kg/m^2^ or higher, whereas adults who are overweight may also be eligible if they have a BMI between 27 and 29.9 kg/m^2^ in conjunction with at least one weight-related comorbidity, such as hypertension, type 2 diabetes, dyslipidemia, cardiovascular disease, or obstructive sleep apnea [112].

Concurrently, semaglutide therapies carry warnings regarding their use in patients with medullary thyroid carcinoma (MTC) or multiple endocrine neoplasia syndrome type 2 (MEN 2) [8,104,108]. Furthermore, during pregnancy and lactation, infant safety in relation to potential drug transfer into human milk must be carefully evaluated, and heightened caution together with close clinical monitoring is required in patients predisposed to pancreatitis, as acute pancreatitis constitutes a recognized serious adverse event associated with this medication [8,102,108,114].

*Tirzepatide*, commercially known as Zepbound for chronic weight management and Mounjaro for type 2 diabetes, is a groundbreaking first-in-class dual agonist that targets both the glucose-dependent insulinotropic polypeptide (GIP) and glucagon-like peptide-1 (GLP-1) receptors [116,117,118,119,120].

Tirzepatide is a 39-amino-acid synthetic peptide that functions as a single-molecule dual agonist for both the GIP and GLP-1 receptors [121]. To enable once-weekly subcutaneous administration, it is structurally modified with a C20 fatty diacid moiety that facilitates albumin binding and significantly prolongs its half-life [122].

Unlike traditional GLP-1 receptor agonists such as semaglutide, tirzepatide exhibits an imbalanced and biased mechanism of action [116,123,124]. It binds to the GIP receptor with an affinity comparable to endogenous GIP, whereas its affinity for the GLP-1 receptor is approximately 5 to 20 times weaker than that of native GLP-1 [122]. At the GLP-1 receptor, it preferentially engages cyclic adenosine monophosphate (cAMP) signaling over β-arrestin recruitment, thereby reducing receptor internalization and degradation, preserving cell surface GLP-1 receptor expression, and sustaining cellular signaling in a way that is believed to optimize metabolic efficacy [122].

This dual-hormone targeting works synergistically. GLP-1 receptor activation slows gastric emptying, promotes satiety, suppresses glucagon secretion, and enhances glucose-dependent insulin secretion, whereas GIP receptor activation further improves insulin sensitivity, increases lipolysis, and stimulates lipid and glucose uptake [121,125,126,127]. The simultaneous activation of both receptors creates a synergistic twincretin effect that surpasses the metabolic outcomes achievable with GLP-1 receptor monotherapy [121,128].

Preclinical models indicate that GIP receptor agonism in the hindbrain, specifically the area postrema, directly suppresses neural activity underlying aversive behaviors, thereby attenuating the nausea and emesis typically caused by robust GLP-1 receptor activation [122]. This interaction facilitates profound appetite suppression alongside improved gastrointestinal tolerability [129].

Unlike GLP-1 receptors, GIP receptors are highly expressed directly on adipocytes [122]. Tirzepatide exploits this feature to alter lipid metabolism by enhancing lipid storage and glucose disposal in white adipose tissue during the fed state and stimulating lipolysis during the fasting state [122]. In addition, dual agonism promotes the emergence of metabolically active beige adipocytes and increases uncoupling protein-1 (UCP1)-mediated thermogenesis, thereby restoring metabolic plasticity and increasing energy expenditure [123].

Tirzepatide also produces robust enhancement of glucose-stimulated insulin secretion and whole-body insulin sensitivity [122]. Whereas GLP-1 inhibits glucagon secretion and GIP can stimulate it during hypoglycemia, tirzepatide balances these effects by reducing fasting glucagon levels overall while preserving a protective counterregulatory glucagon response during hypoglycemic episodes, thereby minimizing the risk of severe hypoglycemia [122].

Beyond regulating weight and glucose, tirzepatide exerts broad cardiometabolic and pleiotropic effects across the cardio-kidney-metabolic system [130]. Its weight-loss efficacy is unprecedented for pharmacotherapy and approaches outcomes typically associated with bariatric surgery [117]. In the landmark SURMOUNT-1 clinical trial, tirzepatide produced a mean weight loss of 20.9% to 22.5% over 72 weeks, and when combined with an intensive lifestyle intervention before medication in the SURMOUNT-3 trial, participants achieved a mean weight loss of 26.6% [123,129].

In patients with T2D, the SURPASS clinical trials demonstrated that tirzepatide provides superior reductions in HbA1c and body weight compared with selective GLP-1 receptor agonists and long-acting insulins [6,131]. It also reduced the risk of progression to T2D by 94% in patients with prediabetes [129,130]. In cardiovascular and metabolic health, tirzepatide significantly improves the lipid profile by lowering triglycerides and LDL cholesterol while increasing HDL cholesterol, and it also reduces blood pressure [6,130].

In the SUMMIT trial, it reduced the risk of cardiovascular death or worsening heart failure events by 38% in patients with obesity and heart failure with preserved ejection fraction (HFpEF) [123,130]. In the SYNERGY-NASH trial, it promoted the resolution of metabolic dysfunction-associated steatohepatitis (MASH) and reduced hepatic fat content [121]. It has also been shown to significantly reduce the apnea–hypopnea index in patients with obstructive sleep apnea (OSA) [129,132].

Consistent with these observations, tirzepatide use is associated with significant reductions in systemic markers of chronic inflammation, including high-sensitivity *C-*reactive protein (hsCRP) and interleukin-6 (IL-6) [124]. Tirzepatide has a generally favorable safety and tolerability profile comparable to that of other incretin-based therapies [131].

The most common adverse events are gastrointestinal, including nausea, diarrhea, vomiting, constipation, and dyspepsia [7,129,132]. These side effects are typically mild to moderate and usually lead to low rates of treatment discontinuation [121,129]. Additionally, tirzepatide is associated with a lower risk of severe hypoglycemia than insulin [7,131].

Tirzepatide is administered as a once-weekly subcutaneous injection and is intended to be used as an adjunct to lifestyle interventions, including a reduced-calorie diet and increased physical activity [129]. Its eligibility criteria vary by indication: for weight management under Zepbound, it is prescribed for adults with obesity (BMI ≥ 30 kg/m^2^) or for those who are overweight (BMI ≥ 27 kg/m^2^) and have at least one weight-related comorbid condition, such as hypertension, cardiovascular disease, elevated cholesterol or dyslipidemia, type 2 diabetes, or obstructive sleep apnea [116,117,129].

For Mounjaro, it is indicated for adults with inadequately controlled type 2 diabetes [116], and it may also be used in adults with obesity and moderate-to-severe obstructive sleep apnea, defined by an apnea–hypopnea index of at least 15 events per hour [132]. However, tirzepatide is restricted in several populations because of safety concerns, including individuals with a personal or family history of medullary thyroid cancer, those with acute or chronic pancreatitis, and women who are pregnant or breastfeeding [7,116]. Additionally, because it delays gastric emptying, it should be used with caution in people with severe gastrointestinal conditions such as gastroparesis [114,126].

### 4.2. Positioning Plant-Based Interventions Within the Incretin-Based Therapies Landscape

Current clinical guidelines recommend intensive lifestyle modifications, consisting of a reduced-calorie diet, increased physical activity, and behavioral therapy, as the first-line treatment for overweight and obesity [8]. However, these interventions often fail to achieve and sustain significant long-term weight loss, and even with rigorous lifestyle changes, mean weight loss typically does not exceed 10% [8,12].

Pharmacotherapy is recommended when patients do not achieve 5% to 10% weight loss after six months of lifestyle intervention [8]. Even among patients who meet strict clinical criteria, major accessibility barriers limit treatment access [12]. High out-of-pocket costs and limited insurance coverage further restrict access, concentrating use among higher socioeconomic groups even though obesity disproportionately affects lower-income populations [5,12]. Rising global demand has also caused supply chain shortages, making these medications difficult to obtain even for patients with prescriptions [12]. Addressing this treatment gap is essential for proactive disease management, because early and accessible intervention may help treat preclinical excess adiposity before it progresses to clinical obesity [1].

Such early treatment may also prevent severe end-organ damage and reduce the risk of life-threatening cardiometabolic complications [1]. To improve accessibility and bridge this gap, researchers are exploring alternative and scalable approaches, including digital health technologies for behavior monitoring, as well as dietary bioactive compounds such as omega-3 polyunsaturated fatty acids and phytochemicals, which may help alleviate obesity-associated inflammation and metabolic dysfunction independently of costly pharmacotherapy [4,17,93].

Although current clinical guidelines reserve anti-obesity pharmacotherapy for individuals with a BMI ≥ 30 kg/m^2^, or a BMI ≥ 27 kg/m^2^ accompanied by at least one weight-related comorbidity such as hypertension, dyslipidemia, or type 2 diabetes, foundational lifestyle modifications, primarily dietary adjustments and physical activity, remain the principal therapeutic approach for those who do not meet these criteria [5,12,113].

To maximize weight loss, clinical models recommend initiating treatment with lifestyle and dietary modifications before introducing pharmacotherapy. However, substantial weight loss often leads to a reduction in lean muscle mass. This loss can adversely affect physical function and lower resting metabolic rate. Hence, preserving fat-free mass during active weight loss is therefore strongly encouraged and maintaining muscle mass also helps prevent mobility limitations and metabolic decline after treatment is discontinued [14,15,42].

As complementary alternatives to conventional medications, which can be cost-prohibitive and carry risks of persistent adverse effects like gastrointestinal complications and metabolic disturbances, natural compounds offer a highly desirable, non-invasive option [4,15,17]. Plant-derived bioactive compounds are widely supported as adjunctive agents to standard lifestyle interventions, physical activity, and medical nutrition therapy [4,13,15].

In certain clinical scenarios, plant-based nutraceuticals even act as effective direct alternatives to some pharmacological therapies [17], with compounds like green tea catechins, resveratrol, and berberine exerting pleiotropic (multi-targeted) mechanisms of action while maintaining highly favorable safety profiles [4,13,15,17].

Evidence from Complementary and Alternative Medicine (CAM) frameworks shows these plant phytoactives can be effectively combined with modern pharmaceutical guidelines to provide a comprehensive, cost-effective treatment strategy [15]. Specifically, plant polyphenols may help counteract the calorie-restriction “energy gap” by naturally promoting anorexigenic hormones and decreasing orexigenic hormones like ghrelin, thus helping to restore the balance between hunger and energy [2].

This holistic phytotherapy appears most effective when combined with precision nutrition and microbiota modulation, such as innovative autologous fecal microbiota transplantation (aFMT) techniques that have shown unique promise [13,29,59]. In one notable trial, patients on a high-polyphenol green-Mediterranean diet who ingested capsules of their own fecal samples, collected at peak weight loss, successfully attenuated expected weight regain and insulin rebound during the post-diet phase [59,60]. Ultimately, incorporating these natural compounds into comprehensive lifestyle interventions offers a promising therapeutic strategy with fewer adverse effects than pharmacological monotherapies, and this combined approach may provide sustainable solutions to global metabolic health challenges [13,17,20].

Within this framework, plant-based strategies and the use of natural bioactive compounds (phytoactives) offer highly effective primary and adjunctive interventions [4,27]. Plant-based dietary patterns, such as vegetarian, vegan, or Mediterranean diets, are effective primary strategies that can yield a 5% to 10% weight reduction in short-term trials while significantly improving cardiovascular risk factors [25,133].

The Green-MED diet is a particularly potent dietary intervention that builds on the Mediterranean diet through calorie restriction, minimal red and processed meat intake, and a greater emphasis on plant-based proteins and polyphenol-rich foods [37,39]. Its main components include daily consumption of green tea, walnuts, and a green shake made from Wolffia globosa (Mankai) [37,59,92]. Greater loss of visceral adipose tissue and intrahepatic fat is independently associated with higher intake of these specific polyphenol-rich plant foods and lower consumption of red meat [37,39].

Certain botanical extracts can also mimic some effects of weight-loss drugs by modulating hunger hormones and improving satiety [4]. For example, a clinical trial using a polyphenolic extract combination of Lippia citriodora (lemon verbena) and *Hibiscus sabdariffa* significantly decreased feelings of hunger and increased subjective satiety and fullness in overweight subjects over a two-month period [2,41].

Polyphenols additionally act as natural prebiotics by modulating the gut microbiota [4,13,15]. Compounds such as resveratrol and green tea catechins have been shown in clinical trials to increase beneficial gut bacteria, including Akkermansia and Bifidobacterium, while suppressing obesity-linked microbes [13,134]. This shift promotes the production of short-chain fatty acids (SCFAs), which improves insulin sensitivity and reduces inflammation [13,14]. Phytoactives can also enhance thermogenesis by promoting the browning of white adipose tissue and upregulating specific uncoupling proteins such as UCP1, while also inhibiting digestive enzymes such as pancreatic lipase and alpha-glucosidase, thereby reducing the absorption of dietary fats and carbohydrates [4,26,88].

Plant-based dietary patterns and selected phytochemicals, especially polyphenols, may also help counter rebound weight gain by acting on appetite regulation, gut microbiome resilience, and energy metabolism [4]. Dietary polyphenols and plant fibers stimulate enteroendocrine cells to release satiety hormones such as GLP-1, peptide YY (PYY), and cholecystokinin (CCK), while also suppressing ghrelin [4]. Green tea catechins, including epigallocatechin-3-gallate (EGCG), and spinach extracts likewise produce satietogenic effects by triggering the rapid release of CCK and GLP-1 [4,15,17].

Dietary polyphenols act as prebiotics to promote beneficial gut bacteria and inhibit pathogens [13,63,134]. These microbial changes increase the production of short-chain fatty acids (SCFAs), including butyrate, propionate, and acetate, and these SCFAs bind to specific intestinal receptors to directly stimulate the natural secretion of GLP-1 and PYY [47,75,134].

This continuous hormone stimulation may help sustain regulation of hunger and glucose homeostasis once pharmacological GLP-1 agonists are withdrawn [16]. These bioactive compounds also inhibit adipogenesis and reduce ectopic fat deposition by downregulating key lipogenic transcription factors, including PPAR-γ and C/EBPα [4,17]. By functioning as multi-targeted metabolic regulators, plant-based diets rich in polyphenols address the homeostatic vulnerabilities exposed when GLP-1 medications are discontinued [2]. Plant-derived bioactive compounds, such as natural polyphenols, therefore represent a highly promising non-pharmacological strategy, as they can naturally stimulate endogenous GLP-1, suppress appetite by favorably modulating hormones such as ghrelin and leptin, alter the gut microbiota, and regulate lipid metabolism by modulating both lipogenesis and lipolysis [4,15].

### 4.3. Limitations and Challenges

Although plant-derived compounds and polyphenols show promise for obesity management, clinical trials remain limited by several recurring problems, including low bioavailability, unfavorable pharmacokinetics, study design constraints, dietary and lifestyle confounding factors, reliance on indirect biomarkers, and missing mechanistic data [15,26,52,68,69]. Most therapeutic compounds are extensively degraded in the liver and gastrointestinal tract, have low absorption rates, and are often eliminated before reaching their target tissues [15,26]. Moreover, many clinical trials include only small numbers of participants and are conducted over short periods, even though obesity requires long-term data to properly evaluate sustained efficacy and safety [26,69]. Additionally, because many studies are free-living trials without strict dietary control, normal differences in daily diet and physical activity introduce substantial confounding variables between individuals [26,52]. Furthermore, many studies rely on indirect biomarkers such as circulating ALT and oxidative stress markers rather than direct tissue assessments, biopsies, and circulating gut metabolites [44,68]. Also, without identifying the precise regulatory networks and metabolic byproduct signaling involved, these interventions remain difficult to fully validate in large-scale clinical studies [16,26].

At the same time, physical activity is consistently highlighted as a cornerstone strategy for managing obesity, cardiovascular disease risk, and metabolic conditions such as non-alcoholic fatty liver disease (NAFLD) and type 2 diabetes [37,52]. Across the provided sources, physical activity is examined both as a prescribed component of comprehensive lifestyle interventions and as a variable that interacts synergistically with dietary polyphenols [22,52]. Many clinical trials mandate or encourage physical activity alongside dietary modifications to achieve metabolic improvements, underscoring its role as a core component of lifestyle interventions.

## 5. Conclusions

Obesity is a chronic, multifactorial disease that has reached epidemic proportions worldwide, driving urgent demand for effective, accessible, and sustainable therapeutic strategies. Incretin-based pharmacotherapies, particularly GLP-1 receptor agonists and dual GIP/GLP-1 agonists, represent the current gold standard for pharmacological weight management, yet their use remains constrained by eligibility criteria, cost, gastrointestinal adverse effects, and rapid weight regain following discontinuation.

This narrative review synthesized clinical evidence from 61 trials and identified plant-derived bioactive compounds as promising adjunctive strategies across three clinically relevant scenarios: for patients ineligible for pharmacotherapy, as complements during drug treatment, and as maintenance strategies following treatment discontinuation. The strongest evidence supports polyphenol-rich dietary patterns, particularly the green-Mediterranean diet, alongside specific extracts including curcumin, bergamot polyphenols, and *Lippia citriodora*/*Hibiscus sabdariffa* combinations. In contrast, isolated high-dose resveratrol and several single-compound interventions demonstrated limited clinical benefit, largely attributable to poor bioavailability. The most effective interventions shared a common feature: pleiotropic mechanisms of action involving AMPK activation, gut microbiota modulation, and appetite hormone regulation.

These findings suggest that an integrated, personalized approach combining incretin-based pharmacotherapy with evidence-based plant-derived interventions may offer a more holistic and sustainable path for obesity management. Future clinical trials should directly evaluate plant-polyphenol combinations alongside GLP-1 receptor agonists to establish synergistic protocols and address the current treatment gap.

## Figures and Tables

**Table 1:** PICO-based inclusion and exclusion criteria for study selection.

PICO Element	Inclusion Criteria	Exclusion Criteria
Population	Adults or adolescents with overweight (BMI ≥ 25 kg/m^2^) or obesity (BMI ≥ 30 kg/m^2^); studies including individuals with metabolic comorbidities (T2DM, MetS, NAFLD) were eligible	Healthy, normal-weight participants as the sole study population
Intervention	Plant-derived compounds, botanical extracts, polyphenol-rich foods, or plant-based dietary patterns administered as primary or adjunctive interventions	Purely pharmacological interventions without a plant-based component
Comparator	Placebo, control diet, or standard care	No comparator required for inclusion, but noted in synthesis
Outcomes	At least one obesity-related endpoint: body weight, BMI, body fat percentage, waist circumference, visceral fat, or associated metabolic markers (fasting glucose, insulin resistance, lipid profile, inflammatory biomarkers)	Studies reporting only in vitro or animal data; studies without extractable outcome data
Study Design	Randomized controlled trials (RCTs); crossover and parallel-arm designs both eligible	Observational studies, case reports, and uncontrolled trials (one observational study [30] was included as supplementary evidence for population-level associations)
Language	English	**-**
Publication Period	January 2012–January 2026	Publications before 2012

**Table 2:** Ranking the efficacy of dietary interventions and plant-derived extracts in the management of obesity.

Ranked	Effectiveness Tier	Treatment/Item	Study Population	Dose/Duration	Key Obesity-Related Effects	Reference(s)
1	HIGH	Green-Mediterranean diet (MED + walnuts + green tea + Mankai)	Adults > 30 y with abdominal obesity/dyslipidemia (*n* = 294)	Walnuts 28 g/d + green tea 3–4 cups/d + Mankai 100 g/d/18 mo	↓ NAFLD prevalence to 31.5%; ↓ intrahepatic fat (−38.9%); ↓ body weight (−3.9 kg); ↓ VAT (−14.1%); ↓ WC; ↓ proinflammatory proteins; ↓ HOMA-IR	[37,38,39]
2	HIGH	Synthetic derivative of lipstatin + Resveratrol + energy-reduced diet	Mexican adults with obesity (BMI 30–39.9)	120 mg synthetic derivative of lipstatin + 100 mg resveratrol × 3/d/6 mo	Greatest weight loss (−6.82 kg, sig. vs. placebo); ↓ BMI, WC, fat mass; ↓ TG, leptin, leptin/adiponectin ratio	[40]
3	HIGH	Calorie-restricted Mediterranean diet (MED) + walnuts	Adults > 30 y with abdominal obesity/dyslipidemia	1200–1800 kcal/d + walnuts 28 g/d/18 mo	↓ NAFLD prevalence to 47.9%; ↓ intrahepatic fat (−19.6%); ↓ body weight (−2.7 kg); ↓ VAT (−6.0%); ↓ WC	[37,39]
4	HIGH	*Lippia citriodora* + *Hibiscus sabdariffa*	Overweight/obese women (BMI 25–35)	500 mg/d/2 mo	↓ Body weight (−3.69 to −4.68 kg); ↓ abdominal circumference (−6.79 cm); ↓ body fat %, BP, heart rate; ↓ hunger, ↑ satiety; ↑ GLP-1	[2,41]
5	HIGH	Bergamot polyphenol extract	Obese metabolic syndrome patients (BMI > 26)	650–1300 mg/d/90 d	↓ Atherogenic index of plasma; ↓ fasting glucose, insulin, HOMA-IR; ↓ body weight and BMI (dose-dependent)	[33]
6	HIGH	Curcumin extract (curcuminoids, 1500 mg/d)	Obese adults with T2DM (BMI ≥ 23)	1500 mg/d/12 mo	↓ Fasting glucose, HbA1c; ↑ HOMA-β; ↓ HOMA-IR; ↓ body weight, BMI; ↑ adiponectin; ↓ leptin	[23]
7	MODERATE	forskolin + green coffee + green tea + beet root + α-lipoic acid + vit E + CoQ10)	Overweight/obese adults (BMI ~30.5)	6 capsules/d/12 wk	↓ Body weight (vs placebo); ↓ total fat mass (DXA); ↓ serum ALT and AST	[42]
8	MODERATE	grapefruit, grape, guarana, green tea, black carrot	Overweight/obese adults (25–55 y)	900 mg/d/16 wk	↓ Body fat mass (incl. trunk fat); ↑ physical activity; ↑ quality of life	[22]
9	MODERATE	Coffee—lightly roasted (Coffea arabica)	Overweight/obese adults (BMI 25–35)	3 cups/d (~1200 mg HCAs)/12 wk	↓ Fat mass (−1.1 kg); ↓ body fat % (−1.4%); ↑ skeletal muscle mass (+0.7 kg)	[43]
10	MODERATE	Caffeine—coffee in adolescents	Adolescents with obesity (12–17 y; BMI ≥ 95th %ile)	160 mg caffeine/d/6 mo	↓ BMI (34.86→31.73); ↓ body fat % (37.4%→32.5%)	[44]
11	MODERATE	*Hibiscus sabdariffa* extract (HSE, 2.7 g/d)	Adults (BMI ≥ 27) with fatty liver	2.7 g/d/12 wk	↓ Body weight, BMI; ↓ serum FFA; improved liver steatosis score	[45]
12	MODERATE	litchi fruit polyphenol	Overweight Japanese adults (BMI 25–30)	200 mg/d/12 wk	↓ Visceral fat area (CT-quantified, sig. vs. placebo); safe	[46]
13	MODERATE	Juçara fruit pulp (lyophilized)	Adults with obesity (BMI 30–39.9)	5 g/d/6 wk	↓ Body fat (kg and %); ↑ HDL-C; ↑ adiponectin (~doubled); ↑ Akkermansia, Bifidobacterium	[47,48]
14	MODERATE	Decaffeinated green tea polyphenols (DGTP)	Girls with obesity (6–10 y)	400 mg/d/12 wk	↓ Body fat %; ↓ serum uric acid; ↓ liver enzymes; no adverse effects	[49,50]
15	MODERATE	Resveratrol + Myoinositol	Obese women with PCOS (20–35 y)	1000 mg RSV + 1000 mg myoinositol 2×/d/12 wk	↓ Body weight, waist-to-hip ratio; ↓ testosterone, insulin; ↑ adiponectin; improved menstrual regularity	[51]
16	MODERATE	Microencapsulated persimmon–karonda + concurrent training	Overweight/obese adults with prediabetes	162 mg/d + training/8 wk	Greatest ↓ in fasting glucose, HbA1c, HOMA-IR; ↓ hs-CRP, IL-6; ↑ adiponectin; ↑ $\dot{V}$O_2_max	[52]
17	MODERATE	Onion peel extract (quercetin)	Overweight/obese Korean adults (BMI > 23)	100 mg quercetin/d/12 wk	↓ Body weight, BMI; ↑ FMD; ↑ EPCs	[53]
18	MODERATE	Cloudy apple juice (polyphenol-rich)	Obese non-diabetic men (BMI > 27)	750 mL/d (~803 mg polyphenols)/4 wk	↓ Body fat %; ↑ lean body mass	[9]
19	MODERATE	EGCG + α-glucosyl hesperidin (green tea + citrus)	Healthy Japanese adults (BMI 23–30)	146 mg EGCG + 178 mg α-gH/12 wk	Prevented body weight gain; ↓ BMI; suppressed visceral fat area increase	[54]
20	MODERATE	Camu-camu polyphenol-rich capsules	Overweight adults with hypertriglyceridemia	1.5 g/d/12 wk	↓ Hepatic fat (MRI; −7.4% vs. +8.4% placebo); ↓ AST, ALT; altered gut microbiota	[55]
21	MODERATE	Rice bran oil (RBO)	Overweight/obese adults with MetSyn (BMI ~31)	30 g/d/8 wk	↓ Total cholesterol, LDL-C; ↑ HDL-C; ↓ fasting glucose; ↓ insulin resistance markers; ↓ MDA	[56]
22	MODERATE	Cinnamon spice (*Cinnamomum burmannii*)	Adults with obesity and prediabetes	4 g/d/4 wk	↓ 24 h glucose; ↓ netAUC glucose; ↓ glucose peak amplitude (continuous monitoring)	[57]
23	MODERATE	Red wine/de-alcoholized red wine polyphenols (Merlot)	Obese MetS patients + healthy controls	272 mL/d (~798 mg total phenols)/30 d	↑ Bifidobacterium, Lactobacillus, butyrate producers; ↓ SBP, DBP, glucose, TG, cholesterol, CRP, LPS; ↑ HDL-C (in MetS)	[58]
24	MODERATE	Autologous fecal microbiota transplant (aFMT) in green-MED	Abdominally obese adults post-weight loss	100 g aFMT capsules/6 mo (months 8–14)	Attenuated weight regain (−0.58 vs. +3.18 kg); attenuated WC gain; attenuated insulin rebound (subgroup-specific)	[59,60]
25	LOW–MODERATE	Whole blackberries	Overweight/obese men (BMI > 25)	600 g/d/7 d	↓ Respiratory quotient (↑ fat oxidation); ↓ insulin AUC; ↓ HOMA-IR	[61]
26	LOW–MODERATE	Elderberry juice (100%)	Overweight/obese adults (BMI > 25)	355 g/d/7 d	↓ Glucose iAUC (−24%); ↑ fat oxidation; beneficial gut microbiota shifts	[62]
27	LOW–MODERATE	Strawberry & cranberry polyphenols	Insulin-resistant overweight/obese adults	333 mg polyphenols/d/6 wk	↑ Insulin sensitivity (clamp); ↓ first-phase insulin secretion; no change in lipids/inflammation	[36]
28	LOW–MODERATE	EGCG + Resveratrol (12 weeks)	Overweight/obese adults	282 mg EGCG + 80 mg RES/12 wk	↑ Skeletal muscle mitochondrial respiration; preserved fat oxidation; ↓ adipose gene expression (no direct weight loss)	[63,64]
29	LOW–MODERATE	Whole blueberries + soluble fiber (pregnancy)	Pregnant women with obesity (high GDM risk)	280 g blueberries + 12 g fiber/d/18 wk	↓ Gestational weight gain; ↓ CRP; ↓ blood glucose post-OGTT	[65]
30	LOW–MODERATE	Microencapsulated persimmon–karonda polyphenol (alone)	Overweight/obese adults with prediabetes	162 mg/d/8 wk	↓ Fasting glucose, HOMA-IR; ↓ hs-CRP, IL-6; ↑ adiponectin (no direct weight loss)	[52]
31	LOW–MODERATE	Hibiscus–inulin beverage	Overweight/obese adults (BMI ≥ 25)	60 mL/d/8 wk	↓ Atherogenic index of plasma; ↓ TyG index	[66]
32	LOW–MODERATE	Whole pecans	Overweight/obese, metabolically at-risk adults	~42.5 g/d/4 wk	↓ Fasting insulin; ↓ HOMA-IR; ↓ composite cardiometabolic risk score	[35]
33	LOW–MODERATE	Diet naturally rich in polyphenols (~2903 mg/d)	Overweight/obese adults at high CVD risk with MetS	~2903 mg total polyphenols/d/8 wk	↓ Fasting and postprandial TG; ↓ urinary 8-isoprostane (oxidative stress)	[67]
34	LOW–MODERATE	Dark sweet cherry drink	Obese adults (BMI 30–40)	400 mL/d/30 d	↓ SBP, DBP; ↓ IFN-γ (pro-inflammatory)	[68]
35	LOW–MODERATE	Concentrated mulberry drink	Adults with obesity	100 g/d/6 wk	↓ SBP, DBP, MAP; ↓ TG; ↓ CRP	[69]
36	LOW–MODERATE	EGCG + Resveratrol (3-day acute)	Healthy overweight adults (BMI 25–30)	282 mg EGCG + 200 mg RES/3 d	↑ Resting and postprandial energy expenditure; ↑ fat oxidation markers	[70]
37	LOW–MODERATE	Healthy dietary guidelines + physical activity (control)	Adults > 30 y with abdominal obesity/dyslipidemia	Standard nutritional counselling + PA/18 mo	↓ NAFLD to 54.8%; modest ↓ intrahepatic fat (−12.2%); ↓ WC; minimal weight loss	[37,39]
38	LOW–MODERATE	Resveratrol (150 mg/d, adipose tissue effects)	Healthy obese men (BMI 28–36)	150 mg/d/30 d	↓ Adipocyte size; ↓ Wnt/Notch signaling; ↑ autophagy; ↓ ACE2 and leptin expression (no weight loss)	[70,71]
39	LOW–MODERATE	Licorice Flavonoid Oil (LFO, 600 mg single dose)	Healthy Japanese females (young)	600 mg single dose (acute)	↓ RER; ↑ fat oxidation during exercise; ↑ VO_2_	[72]
40	LOW–MODERATE	Dietary flavonoids (observational, habitual intake)	Italian adults (n = 1937; cross-sectional)	Highest quartile (~455 mg/d habitual intake)	Inverse association with excess body weight (OR 0.66); ↓ obesity odds with catechins (OR 0.33)	[30]
41	LOW	Whole Gala apples (inflammation focus)	Overweight/obese adults (BMI ~33)	3 apples/d/6 wk	↓ CRP (−17%); ↓ IL-6 (−12%); ↓ LPS-binding protein (no weight/fat change)	[34]
42	LOW	Freeze-dried strawberry powder (arthritis focus)	Obese adults with knee osteoarthritis	50 g/d/12 wk	↓ IL-6, IL-1β, MMP-3; ↓ pain scores (arthritis outcomes, not weight/fat)	[73]
43	LOW	tomato extract	Overweight/obese adults (BMI 28–35)	300 mg/d/4 wk	↓ Plasma TMAO, LPS; modulated gut microbiota (no weight/fat/glucose effects)	[74]
44	LOW	inulin + oat β-glucan + blueberry polyphenols	Overweight/obese adults with elevated fasting glucose	~8.8 g fiber + 724 mg polyphenolics 2×/d/4 wk	Improved OGTT glucose tolerance; ↑ satiety (no direct weight/fat loss)	[75]
45	LOW	Whole-grain wheat	Overweight/obese sedentary adults	70 g/d/8 wk	↑ Ferulic acid metabolites; ↓ TNF-α (biomarker only)	[76]
46	LOW	Pigmented rice (purple and red varieties, acute)	Obese sedentary adults (BMI > 30)	1 cup cooked/single acute dose	↑ Antioxidant activity; ↓ MDA; ↓ inflammatory cytokines (acute, no weight)	[77]
47	LOW	Pelemir (*Cephalaria syriaca*) flour bread	Healthy, obese, and T2DM adults	0.3% pelemir flour/single acute meal	↑ Postprandial insulin and GLP-1 iAUC; no change in glucose (acute incretin effect only)	[78]
48	LOW	Resveratrol + Curcumin (acute single dose)	Older adults with abdominal obesity	200 mg RSV + 100 mg curcumin/single dose	↓ Postprandial sVCAM-1; no change in CRP, IL-6, IL-8, MCP-1	[79]
49	LOW	*Ascophyllum nodosum* seaweed extract	Overweight/obese adults (BMI ≥ 25)	100 mg polyphenols/d/8 wk	↓ DNA damage in obese subgroup; no change in oxidative capacity or CRP	[80]
50	LOW	Melinjo Seed Extract (MSE)	Healthy young Japanese males	150–300 mg/d/14 d	↑ HMW/total adiponectin ratio (300 mg; genotype-dependent); no sig. diff. vs. placebo	[81]
51	NULL/NEGATIVE	Cranberry extract beverage (low-energy)	Obese adults with elevated glucose (BMI 30–45)	450 mL/d/8 wk	No change in insulin sensitivity; ↓ 8-isoprostane; ↓ TAG in high-CRP subgroup only	[82]
52	NULL/NEGATIVE	Red wine polyphenol extract	Obese adults (BMI ≥ 30)	600 mg/d/8 wk	No change in insulin sensitivity, glucose, insulin, or lipid parameters	[83]
53	NULL/NEGATIVE	Blueberry powder (older adults)	Overweight/obese sedentary older adults (>60 y)	36 g/d/12 wk	No overall microbiome shift; ↓ HDL-P and ApoA-I (potentially unfavorable)	[84]
54	NULL/NEGATIVE	Resveratrol (75 mg/d, non-obese postmenopausal women)	Non-obese postmenopausal women	75 mg/d/12 wk	No change in body composition, RMR, lipids, inflammation, or insulin sensitivity	[85]
55	NULL/NEGATIVE	Grape/pomegranate pomace (acute)	Adults with abdominal obesity (40–60 y)	10 g/single acute dose	No change in glucose, insulin, or antioxidant capacity during OGTT	[86]
56	NULL/NEGATIVE	*Aronia melanocarpa* (chokeberry) juice	Adults at cardiovascular risk	100 mL/d/4 wk	No ↓ cholesterol, LDL, or BP vs. placebo; altered fatty acid profile only	[87]
57	NULL/NEGATIVE	*Hibiscus sabdariffa* extract (1000 mg/d, Thailand)	Adults with central obesity and mild MetS	1000 mg/d/12 wk	No effects on HOMA-IR, glycemia, BMI, WC, lipids, or BP vs. placebo	[88]
58	NULL/NEGATIVE	Rutin (capsule or yoghurt)	Overweight adults (without diabetes)	500 mg/d/12 wk	No change in β-cell response, glycemic endpoints, or gut microbiota	[31]
59	NULL/NEGATIVE	Resveratrol (1.5 g/d, NAFLD)	Overweight adults with biopsy-verified NAFLD	1.5 g/d/6 mo	No ↓ ALT vs. placebo; no histological improvement; adverse event reported	[89,90]
60	NULL/NEGATIVE	Grape polyphenol powder (4-week)	Obese, healthy adults	60 g/d/4 wk	No change in glucose, insulin, TG, or inflammation after 4 wk; ↑ sVCAM-1	[32]
61	NEGATIVE	Resveratrol (3000 mg/d, NAFLD)	Overweight/obese men with NAFLD	3000 mg/d/8 wk	No ↓ insulin resistance, hepatic steatosis, or abdominal fat; ↑ ALT and AST (hepatic stress)	[91]

The upward and downward arrows indicate an increase and a decrease, respectively, in the corresponding parameter. Abbreviations: ACE2, angiotensin-converting enzyme 2; aFMT, autologous fecal microbiota transplant; α-gH, α-glucosyl hesperidin; ALT, alanine aminotransferase; ApoA-I, apolipoprotein A-I; AST, aspartate aminotransferase; AUC, area under the curve; BMI, body mass index; BP, blood pressure; CoQ10, coenzyme Q10; CRP, *C-*reactive protein; CT, computed tomography; CVD, cardiovascular disease; DBP, diastolic blood pressure; DGTP, decaffeinated green tea polyphenols; DXA, dual-energy X-ray absorptiometry; EGCG, epigallocatechin gallate; EPCs, endothelial progenitor cells; FFA, free fatty acids; FMD, flow-mediated dilation; GDM, gestational diabetes mellitus; GLP-1, glucagon-like peptide-1; HbA1c, glycated hemoglobin; HCAs, hydroxycinnamic acids; HDL-C, high-density lipoprotein cholesterol; HDL-P, high-density lipoprotein particle number; HMW, high molecular weight; HOMA-β, homeostatic model assessment of β-cell function; HOMA-IR, homeostatic model assessment of insulin resistance; hs-CRP, high-sensitivity *C-*reactive protein; HSE, *Hibiscus sabdariffa* extract; iAUC, incremental area under the curve; IFN-γ, interferon-γ; IL-1β, interleukin-1β; IL-6, interleukin-6; IL-8, interleukin-8; LDL-C, low-density lipoprotein cholesterol; LFO, licorice flavonoid oil; LPS, lipopolysaccharide; MAP, mean arterial pressure; MCP-1, monocyte chemoattractant protein-1; MDA, malondialdehyde; MED, Mediterranean diet; MetS (MetSyn), metabolic syndrome; MMP-3, matrix metalloproteinase-3; MRI, magnetic resonance imaging; MSE, melinjo seed extract; NAFLD, non-alcoholic fatty liver disease; netAUC, net area under the curve; OGTT, oral glucose tolerance test; OR, odds ratio; PA, physical activity; PCOS, polycystic ovary syndrome; RBO, rice bran oil; RER, respiratory exchange ratio; RES (RSV), resveratrol; RMR, resting metabolic rate; SBP, systolic blood pressure; sVCAM-1, soluble vascular cell adhesion molecule-1; T2DM, type 2 diabetes mellitus; TAG, triacylglycerol; TG, triglycerides; TMAO, trimethylamine N-oxide; TNF-α, tumor necrosis factor-α; TyG index, triglyceride-glucose index; VAT, visceral adipose tissue; VO_2_, oxygen consumption; VO_2_max, maximal oxygen consumption; WC, waist circumference.

## Data Availability

No new data were created or analyzed in this study. Data sharing is not applicable to this article.
